# From photoisomerization to logic operations: a theoretical study of pseudo-hemiindigo derivatives

**DOI:** 10.3389/fchem.2026.1962171

**Published:** 2026-10-14

**Authors:** Christina Eleftheria Tzeliou, Demeter Tzeli

**Affiliations:** 1 Laboratory of Physical Chemistry, Department of Chemistry, National and Kapodistrian University of Athens, Athens, Greece; 2 Theoretical and Physical Chemistry Institute, National Hellenic Research Foundation, Athens, Greece

**Keywords:** absorption spectra, DFT, fluorescence spectra, hemiindigo, molecular logic gates, photoisomerization, photoswitches, transition states

## Abstract

Over the last decades the hemiindigo photoswitches, derived from indigo dye, have gained attention because they uniquely absorb visible light in both isomeric states, making them suitable when high-energy UV light is undesirable. Here, the photophysical properties of a series of pseudo-hemiindigo derivatives with anthracene, naphthalene, phenanthrene, pyrene, and pyrrole, i.e., **1-L**, L = An, Na, Ph, Pn and Pl, are studied via Density Functional Theory (PBE0, B3LYP, M06-2X, ωB97XD and CAM-B3LYP) calculations. Their cis-cis, cis-trans, and trans-trans isomerization processes are investigated as well as their effect on the absorption and fluorescence spectra of the molecules. The isomerization process affects the absorption and fluorescence spectra, i.e., shifts of up to 30 nm and 70 nm are observed, respectively while the transition state is shifted up to about 150 nm. Irradiation with light of 580–620 nm can induce photoisomerization around double bonds converting between cis and trans configurations depending on the L group. It is found that all have the potential to be used as two-input molecular logic gates, where two subsequent irradiations are used as inputs. Emission outputs at about 420, 450, 480, 540, 560, 630, 780 nm can be utilized as output to build AND and Converse Nonimplication logic gates.

## 1 Introduction

Molecular logic gates (MLGs) were introduced in 1993, however several receptor molecules that performed basic logic-like operations, linking ionic or molecular-recognition events to photonic (fluorescence) signals, had already been reported (de Silva and D Rupasinghe, 1985; Huston et al., 1989; Hosseini et al., 1990; Van Arman and Czarnik, 1993). In those systems, a chemical binding event “input” altered fluorescence intensity “output”. Photo-induced electron transfer, recognized as a key photochemical mechanism, was applied by de Silva et al., in 1985 to improve fluorescent pH indicators. Since 1993, when the first MLG was synthesized (de Silva et al., 1993), the field has been thoroughly studied ever since, determining the crucial factors for a molecule to act as an MLG. It has been proved that molecules respond to changes in their environment (Erbas-Cakmak et al., 2018). The presence of various ions, neutral species, pH, temperature, and viscosity, among others, result in color or emission changes due to the complex interaction of numerous excited state processes and environmental variables. Different combinations of inputs can lead to different outputs.

Adolf von Baeyer was the first to describe and synthesize hemiindigo (HI) in 1883 (Baeyer, 1883). It presents exceptional thermal bistability and excellent switching performance in the visible part of the electromagnetic spectrum rendering it a distinctive candidate for use in medicine and material science. It can be easily obtained and can be synthesized through condensation reactions between benzaldehyde derivatives and indoxyl acetates (Bartelmann and Dube, 2022).

Katritzky et al. (1988) demonstrated the occurrence of mutual photoisomerization between the Z- and E-isomers in the hemiindigo dye 2-(2-pyrrolylidene)indolin-3-one, which has a pyrrole ring and can form an intramolecular hydrogen bond in the E-isomer, upon color change in benzene from greenish yellow to reddish orange. Ikegami and Arai (2005) then reported on the fluorescence and isomerization behavior of 2-(2-pyrrolylidene)indolin-3-one in ethanol and water, with fluorescence emission of E-isomer being highly dependent on both solvent and temperature, while the fluorescence emission was not detected in an aqueous solution. Following, an experimental and theoretical study by Petermayer et al. (2017) illustrated the red-shifted absorption of hemiindigo in the visible spectrum and the use of blue or green and red light to achieve almost perfect photoswitching. Petermayer and Dube (2018a) then investigated theoretically five distinct HI derivatives with n-propyl or p-tolyl groups added at the indoxyl nitrogen atom or no further substitution at all, that act as favorable photoswitches for practical use due to their distinctive properties, along with their functionalizability and synthetic feasibility. Afterwards, they investigated axially chiral hemiindigo photoswitches that upon exposure to visible light present peculiar chiroptical alterations (Petermayer and Dube, 2018b). Photoswitching does not lead to complete stereo-configuration alteration of their chiral axes, showing the strong modulation of the entire electronic circular dichroism spectrum due to the different chemical twists in the Z and E isomeric switching states.

Zhao and Yang (2020) studied both experimentally and theoretically the fluorescence and photoisomerization properties of HI in dioxane and methanol, finding adequately quenching of the fluorescence of HI in methanol, but strong in dioxane. The fluorescence quenching of HI in methanol is a result of hydrogen bonds strengthening between solutes and solvents in the excited state. A study by Sacherer, Hampel, and Dube (Sac et al., 2023) demonstrated four-state switching, chemical fueling, and reversible inscription into transparent polymers using green and red light and acid/base stimuli with an extensive photochemical analysis of all compounds to highlight the prospect of these distinct light-responsive molecular tools. In 2021, Quintana-Romero and Ariza-Castolo (2021) studied experimentally six stable pseudo-hemi-indigo-derived MLGs capable of solving complex logic operations. The MLG design here relies on a molecular switch that experiences Z and E isomerism, thus the molecular switch axis has to be a double bond. The HI structure was preferred due to its interaction with visible light, while Z and E pseudo-hemiindigo isomers can be utilized for selective isomerization since they have distinct absorption spectra. These MLGs could be used as chemical sensors or as particular intracellular indicators. According to Zhao and Yang (2020), this particular kind of MLG with two inputs may be used as a real logic gate.

More recently, azobenzene based molecular monolayers were studied by Li et al. (2025) who compared the dual molecular switching functionality with light or protonation and proposed an azobenzene-based device that uses a dual switching protocol and could be used to produce an “AND” logic gate based on monolayer (photo)chemistry. Das et al. (2024) showed that appropriate selection of external inputs can lead to “on−off” and “off−on” emission switching in the NIR region. Findings showed luminescence in the NIR region and substantial absorption across the UV−visible area. Exposure to visible light resulted in trans → cis and trans−trans → trans−cis isomerization of the bridging ligands, while exposure to UV light leads to reverse isomerization. Photoswitching was found to occur faster when acid is present.

Then, the development of two redox-switchable chiral metal-organic cages that display an on-off chirality logic gate upon redox transitions that modify their chemical and stereostructural dynamics was reported by Lu et al. (2024). These complexes can be used to accomplish stereoselective identification and transformation that can be monitored by spectroscopic analysis. Finally, Gernet et al. (2025) conducted a detailed review on the developments in the chemistry and applications of hemiindigoid photoswitches, where they highlight that while HI was first synthesized over a century ago, only recently it started attracting more interest, mostly due to its potential in photopharmacological applications. It is their simple synthesis, convenient accessibility and the ability to isomerize at red-shifted wavelengths that render HIs promising candidates for photoswitching applications in various fields of chemistry.

Overall, hemiindigo derivatives represent a versatile and promising platform for innovation across materials science, and biology. It is essential to further develop and expand their applications. In the present study, the photophysical properties of a series of pseudo-hemiindigo derivatives are studied via Density Functional Theory (DFT) calculations. The main aims of this work are: (i) the investigation of the isomerization processes, (ii) the study of the photophysical properties of both isomers and their transition states of the molecules, and (iii) the exploration of their potential as MLG and/or photoswitches.

Overall, the present study provides detailed insight into the isomerization processes, including the electronic and photophysical properties not only of the isomers but also of the transition states resulting from rotation around the double and single bonds. To the best of our knowledge, no such comprehensive study has been reported. Experimental data are available for only one of the five molecules investigated, specifically for the pseudo-hemiindigo pyrrole derivative, for which only the absorption and emission spectra of the trans and cis isomers have been reported. Data are also available for an isomer of the calculated pseudo-hemiindigo anthracene derivative. In the present study, the outer ring of anthracene is attached to the pseudo-hemiindigo unit, whereas in Quintana-Romero and Ariza-Castolo (2021), anthracene is attached through its central ring. Finally, Boolean logic is employed to evaluate the potential application of these systems as molecular logic gates (MLGs). In Quintana-Romero and Ariza-Castolo (2021), the suitability of the pseudo-hemiindigo pyrrole derivative as an MLG, based on isomerization and protonation, was investigated. In the present study, the potential of all five molecules to function as two-input molecular logic gates is examined, with two successive irradiations serving as the inputs. Thus, the present study makes a significant contribution to existing literature.

## 2 Computational details

The pseudo-hemiindigo molecules, **1-L**, where L = anthracene, phenanthrene, naphthalene, pyrrole and pyrene, have been studied via DFT and Time-Dependent DFT (TD-DFT), Scheme 1. At first, conformational analyses were carried out, where all species were fully energetically optimized in the ground state to calculate the trans, cis and transition states for each species. Regarding the notation of the molecules, **1-L**, the abbreviations “An”, “Ph”, “Na”, “Pl” and “Pn” are used for anthracene, phenanthrene, naphthalene, pyrrole and pyrene, respectively. The cis structure is stated with the index “*c*”, while trans with “*t*”. Transition states are stated with the superscript “*ts*” and isomerizations are stated with subscripts as follows: “*ct”* stands for cis → trans, “*cc”* for cis → cis and “*tt”* for trans → trans, see Scheme 1.

**SCHEME 1:**
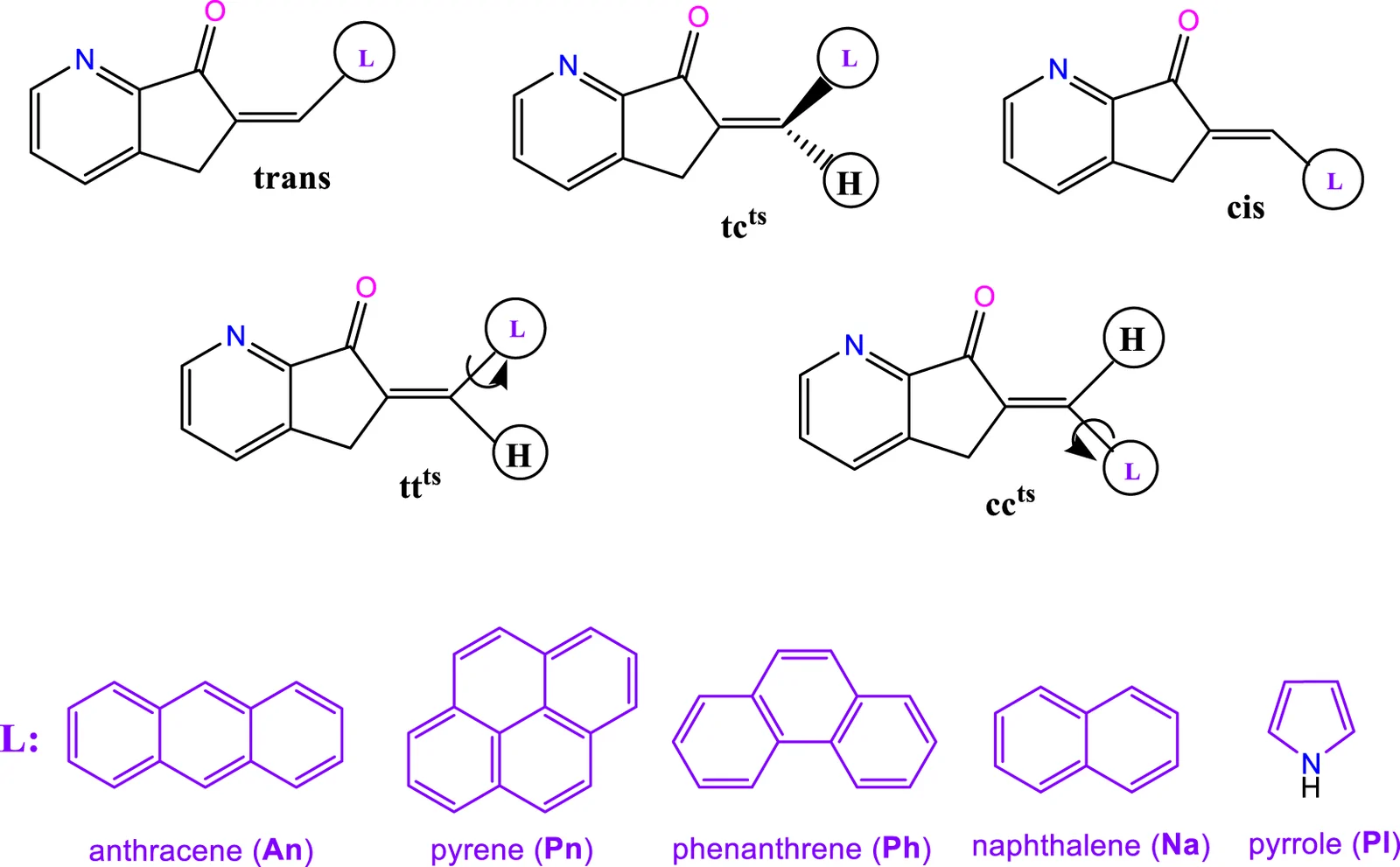
General scheme of the structures under study, where **L** is *anthracene*, *phenanthrene*, *naphthalene*, *pyrrole* and *pyrene*.

Previous theoretical studies have shown that both PBE0 and Β3LYP functionals work efficiently for the photophysical study of molecules including fluorophores, see for instance Refs (Kolokytha et al., 2025; Guido et al., 2013; Jacquemin et al., 2012; Tzeli et al., 2016; Tzeliou and Tzeli, 2022; Morgante and Peverati, 2023; Tzeliou and Tzeli, 2023; Papamichalis et al., 2025). However, to determine the best methodology for the present work, a systematic study of the trans and cis isomers of **1-L**, where L = pyrrole and anthracene was carried out, since there were available experimental data for the **1-Pl** molecule and for an isomer of **1-An**, i.e., in the present study, the outer ring of anthracene is attached to the pseudo-hemiindigo unit, whereas in Quintana-Romero and Ariza-Castolo (2021), anthracene is attached through its central ring of these molecules (Quintana-Romero and Ariza-Castolo, 2021). Specifically, the PBE0 (Adamo and Barone, 1999; Perdew et al., 1996), B3LYP (Becke, 1993), M06-2X (Zhao and Truhlar, 2008), ωB97XD (Chai and Head-Gordon, 2008) and CAM-B3LYP (Yanai et al., 2004) functionals in conjunction with 6-31G (d, p) (Hehre et al., 1972), 6-311G+(d, p) (McLean and Chandler, 1980; Raghavachari et al., 1980) and def2-TZVP (Weigend et al., 2005; Weigend, 2006) basis sets were applied. Evaluation of the results showed that PBE0 functional yielded the best agreement with the experimental data, see Section 3.1, as it has been concluded in previous studies (Guido et al., 2013; Jacquemin et al., 2012; Tzeli et al., 2016; Tzeliou and Tzeli, 2022; Morgante and Peverati, 2023; Tzeliou and Tzeli, 2023; Papamichalis et al., 2025; Improta et al., 2009, Zhao et al., 2020); thus, PBE0 was used for the calculation of all molecules.

The studied molecules were optimized in both cis- and trans-conformations at the PBE0/6-31G (d,p) and PBE0/def2-TZVP level of theory. The absorption and emission spectra of the structures were calculated at the PBE0/def2-TZVP level of theory. In all cases, the absorption spectra of the studied systems were calculated including up to 50 singlet- and triplet-spin excited electronic states. Transition states (cis → cis, cis → trans, and trans → trans) were obtained, see Figure 1, as well as their corresponding absorption spectra. Finally, emission spectra were also obtained for the first singlet state of each molecule in both cis and trans-conformations in the PBE0/def2-TZVP level of theory as well.

**FIGURE 1:**
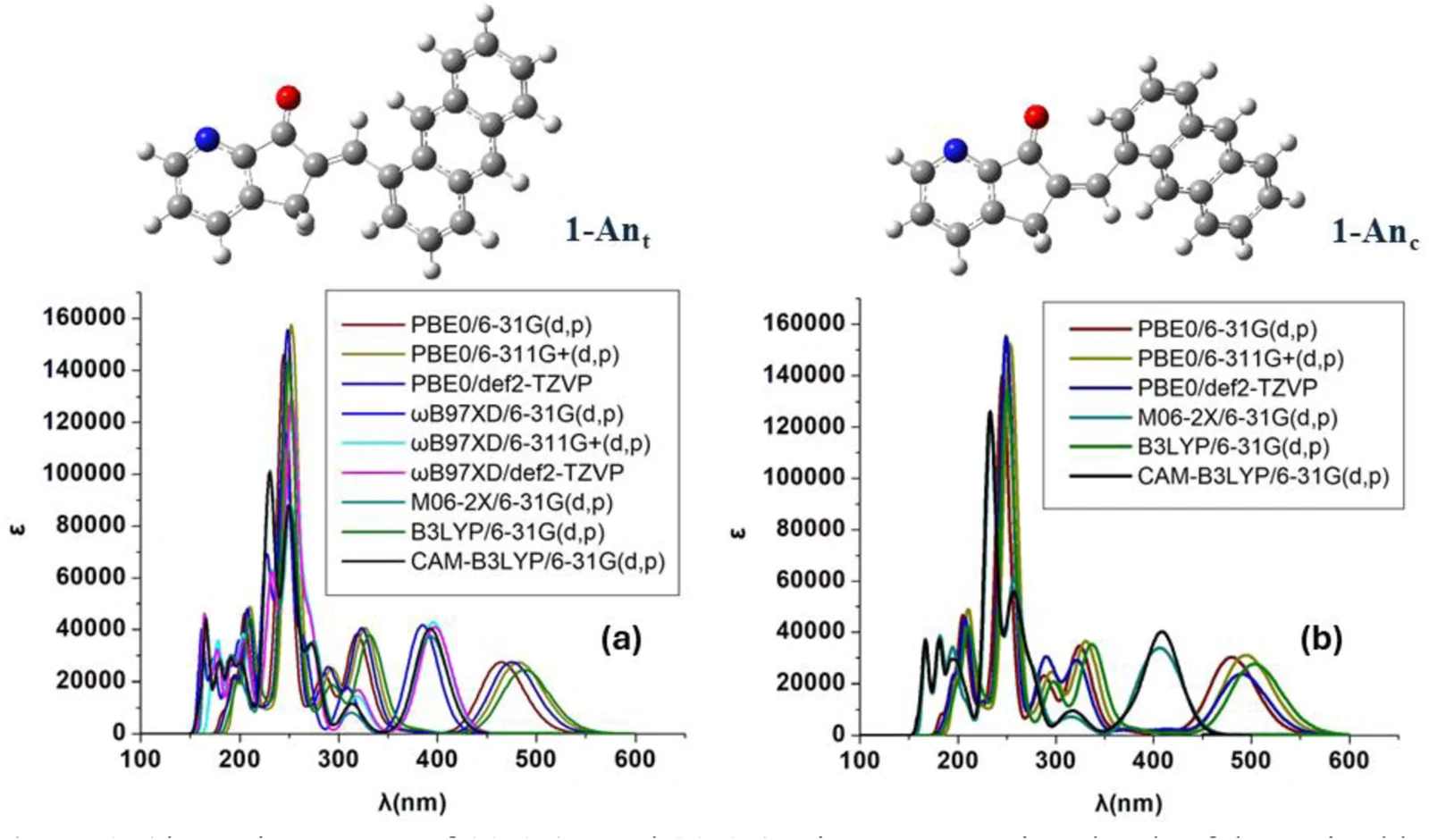
Absorption spectra of **(a) 1**-An_t_ and **(b) 1**-An_c_ isomers at various levels of theory in chloroform solvent.

The transition states (ts) were calculated employing the STQN method for locating transition structures (Peng et al., 1996). For all calculated molecules, frequences were computed to verify that the computed structures are true minimum structures or transition states. All studies were performed in chloroform solvent that was added as a dielectric constant and thus, PCM (Miertuš et al., 1981; Tomas et al., 2005) was also employed. All calculations were carried out with the Gaussian16 code (Frisch et al., 2016).

## 3 Results and discussion

### 3.1 DFT analysis

A systematic DFT investigation was carried to out to calculate the absorption spectra of the cis- and trans-conformers of **1** with pyrrole and anthracene, see Figures 1, 2, via a series of DFT methodologies to find the best methodology for the present study. DFT calculations were accrued out using the PBE0, B3LYP, M06-2X, ωB97XD and CAM-B3LYP functionals, and the 6-31G (d,p), 6-311G+(d,p) and def2-TZVP basis sets. The first main absorption peaks with available experimental data are given in Table 1, while the calculated UV-vis absorption spectra of both trans and cis **1**-An and **1**-Pl molecules are depicted in Figures 1, 2.

**FIGURE 2:**
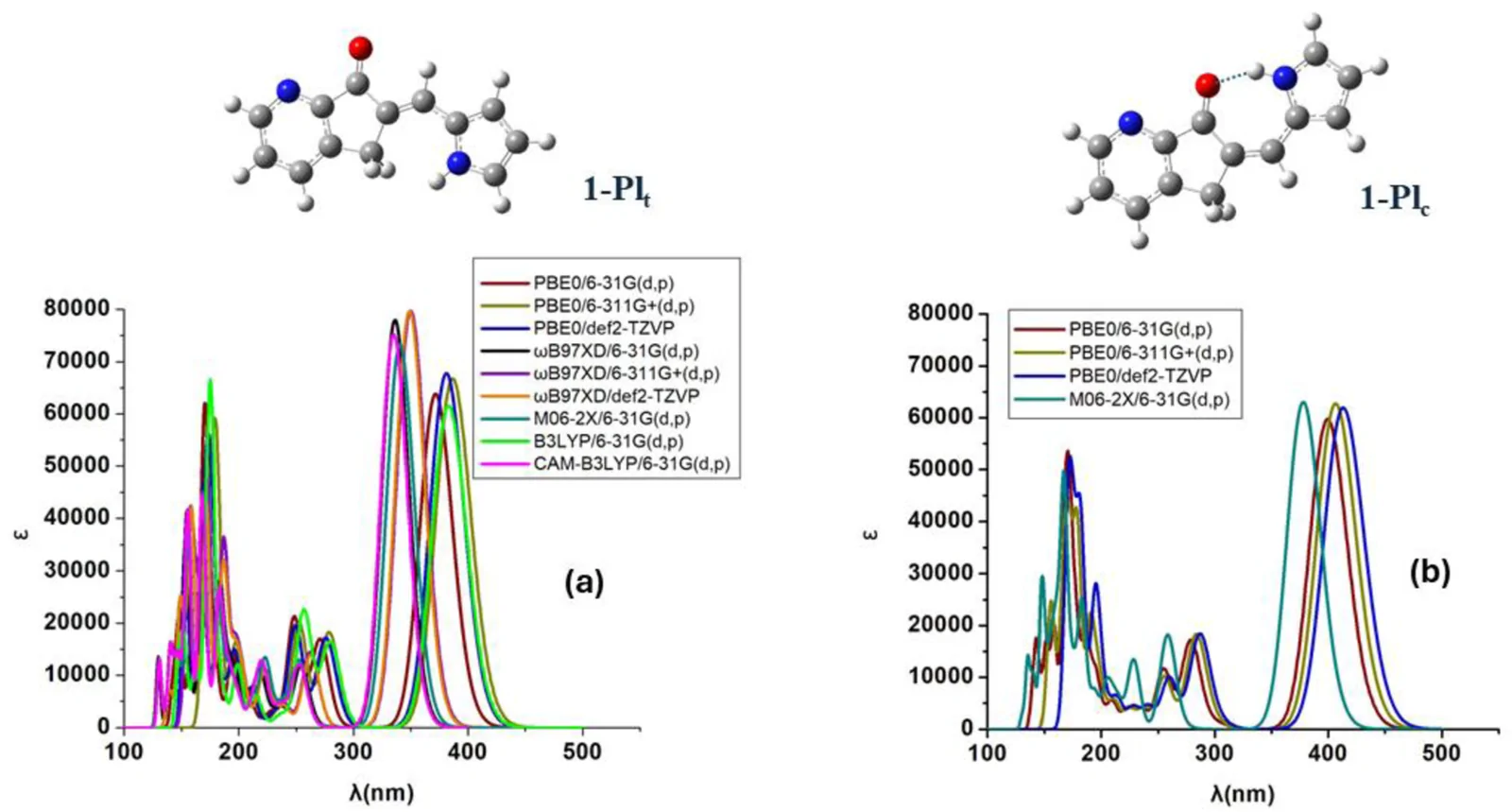
Absorption spectra of **(a) 1**-Pl_t_ and **(b) 1**-Pl_c_ isomers at various levels of theory in chloroform solvent.

**TABLE 1:** Calculated main vis absorption peaks λ in nm of **1**-An_t_ and **1**-Pl_t_ using various methodologies in chloroform solvent; the deviation in nm from available experimental values are also included.

Methodology	1-An_t_ ^a^	Deviation	1-Pl_t_ ^b^	Deviation
PBE0/6-31G (d,p)	464.5	18.5	387.8	−12.2
PBE0/6-311G+(d,p)	482.0	36.0	386.7	−13.3
PBE0/def2-TZVP	462.3	16.3	391.7	−8.3
ωB97XD/6-31G (d,p)	385.2	−60.8	358.5	−41.5
ωB97XD/6-311G+(d,p)	395.8	−50.2	357.2	−42.8
ωB97XD/def2-TZVP	397.1	−48.9	360.1	−39.9
M06–2X/6-31G (d,p)	394.5	−51.5	372.0	−28.0
B3LYP/6-31G (d,p)	488.1	42.1	400.1	+0.1
CAM-B3LYP/6-31G (d,p)	392.7	−53.3	355.6	−44.4
Expt^c^	(446.0)^d^	​	400.0	​

^a^ ∼0.995 |H → L>.

^b^ ∼0.996 |H → L>.

^c^ Quintana-Romero and Ariza-Castolo (2021).

^d^ In **1-An**_**t**_, the outer ring of anthracene is attached to the pseudo-hemiindigo unit (**1**), whereas in Quintana-Romero and Ariza-Castolo (2021), the central ring of anthracene is attached to **1**.

It is found that the general shape of the calculated UV-vis absorption spectra is similar for all different methodologies. However, the methodologies are divided into two groups. One group contains the PBE0 and B3LYP functionals and the other one the ωΒ97XD, M06-2X and CAM-B3LYP functionals. The absorption spectra calculated for the first group are red shifted with respect to the absorption spectra of the second group by about 70 nm and 30 nm for the **1-An**_**t**_ and **1-Pl**_**t**_ respectively in the vis and near-UV area.

Comparing the calculated main vis absorption peak with the experimental one, it is found that the deviation of the calculated peaks from the experimental ones, applying all functionals, ranges from 0 nm to 61 nm. The smallest deviation is observed for the PBE0/def2-TZVP method. Specifically, for the **1**-An_t_ molecule, the PBE0/def2-TZVP main vis peak is calculated at 462.3 nm. Experimentally, the main absorption peak for the hemi-indigo derivative where anthracene is attached through its central ring has been measured at 446 nm (Quintana-Romero and Ariza-Castolo, 2021), however small differences are reasonable since the anthracene is attached from a different ring to hemi-indigo.

For the **1**-Pl_t_ molecule, the PBE0/def2-TZVP main vis peak is calculated at 391.7 nm, i.e., it is blue shifted only by 8.3 nm (0.06 eV) with respect to the experimental value of 400 nm (Quintana-Romero and Ariza-Castolo, 2021). It should be noted that the first peak exhibits a small charge-transfer character, as discussed below, for all functionals. This indicates that the choice of the functional affects the relative position of the peak but not its character. Furthermore, this peak corresponds to a $|H\to L\rangle$ excitation (see Table 1). Two additional absorption peaks are calculated in the 200–300 nm region. For the PBE0 and B3LYP functionals, the first UV absorption peak appears at 271–278 nm, whereas the second occurs at 248–253 nm, depending on the basis set used. For the M06–2X and ω B97XD functionals, the corresponding peaks are predicted at 252–263 nm and 223–239 nm, respectively, depending on the basis set. Thus, the M06–2X and ω B97XD functionals predict UV absorption peaks that are blue-shifted by approximately 20 nm relative to the corresponding peaks obtained with the PBE0 and B3LYP functionals. This shift has been observed in other similar molecules (Morgante and Peverati, 2023; Tzeliou and Tzeli, 2023).

### 3.2 Geometry

The calculated structures of the **1-An**, **1-Na**, **1-Ph**, **1-Pl**, and **1-Pn** molecules along with the absorption spectra are depicted in Figures 3–7. Selected geometries are given in Table 2. The trans (**1-L**_**t**_) and the cis (**1-L**_**c**_) isomers were calculated as well as the transition states of the trans-cis isomerization (**1-L**_**ct**_^**ts**^), the transition states regarding the rotation of the L group along the C-L single bond for both cis isomers (**1-L**_**cc**_^**ts**^) and trans isomers (**1-L**_**tt**_^**ts**^), see Scheme 1.

**FIGURE 3:**
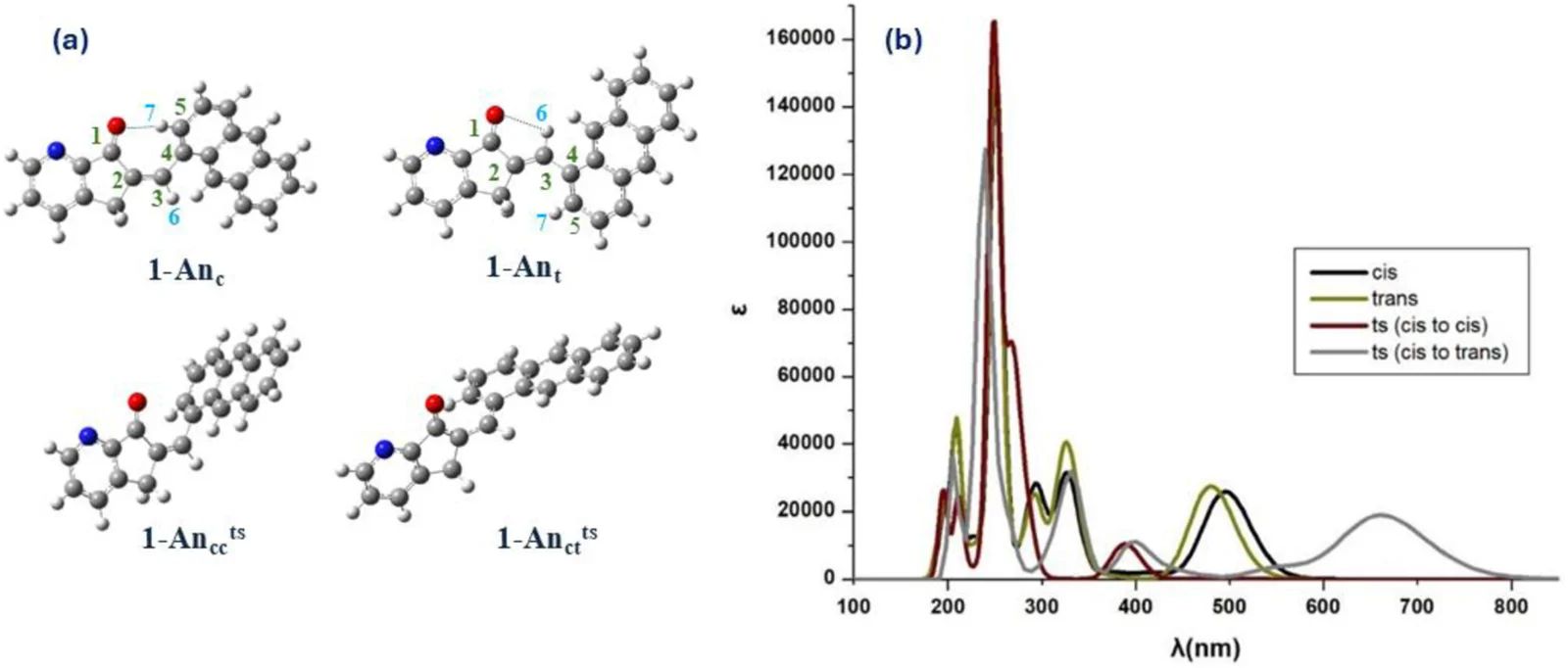
**(a)** Optimized *cis-, trans-* and transition states of **1-An (b)** absorption spectra of **1**-**An** at the PBE0/def2-TZVP level of theory in chloroform.

**FIGURE 4:**
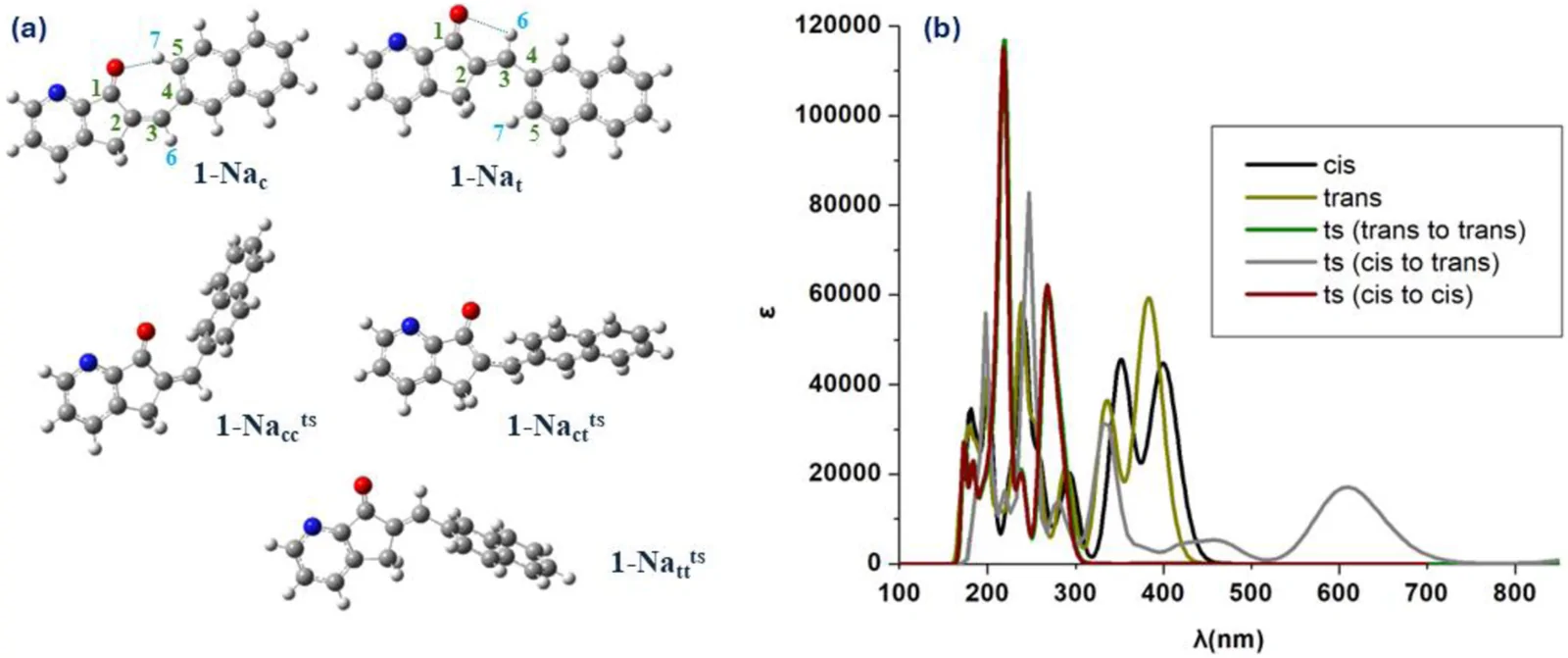
**(a)** Optimized *cis-, trans-* and transition states of **1-Na (b)** absorption spectra of **1**-**Na** at the PBE0/def2-TZVP level of theory in chloroform.

**FIGURE 5:**
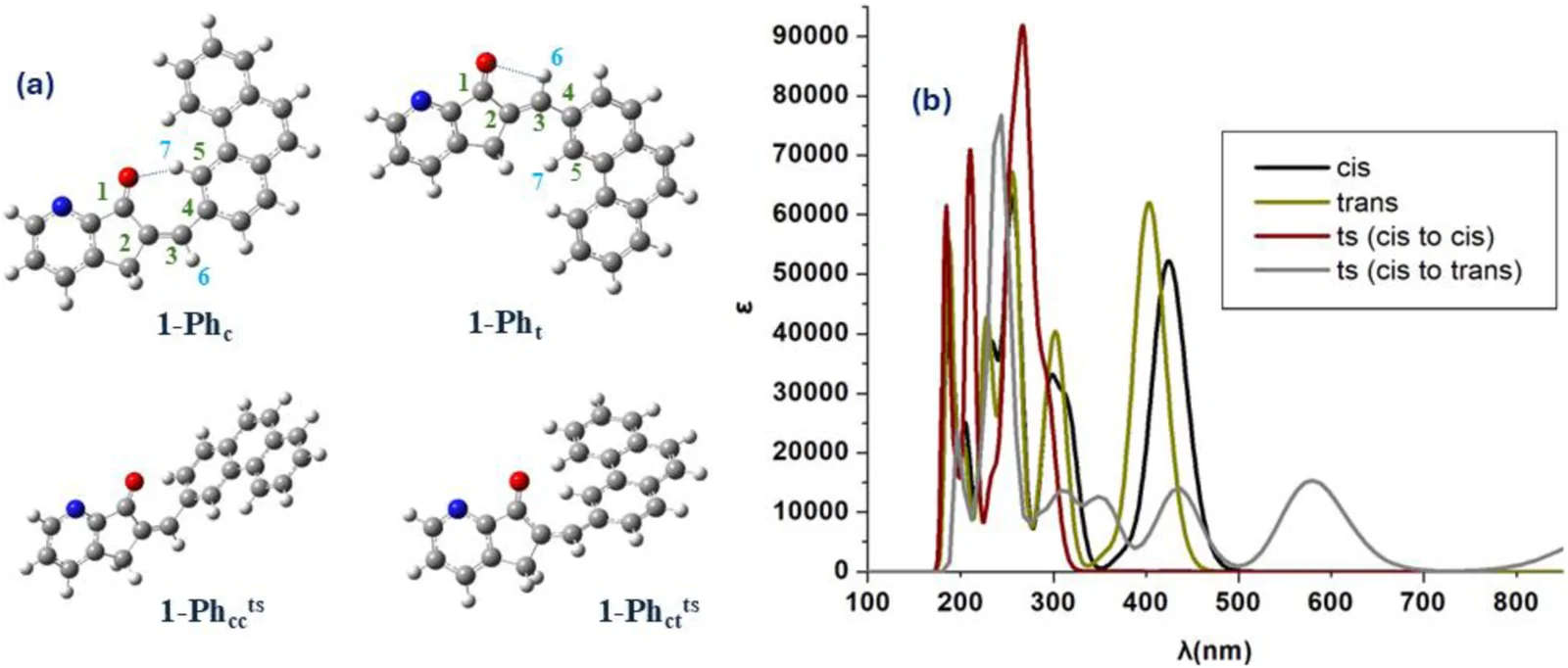
**(a)** Optimized *cis-, trans-* and transition states of the **1-Ph** molecule **(b)** absorption spectra of **1**-**Ph** at the PBE0/def2-TZVP level of theory in chloroform.

**FIGURE 6:**
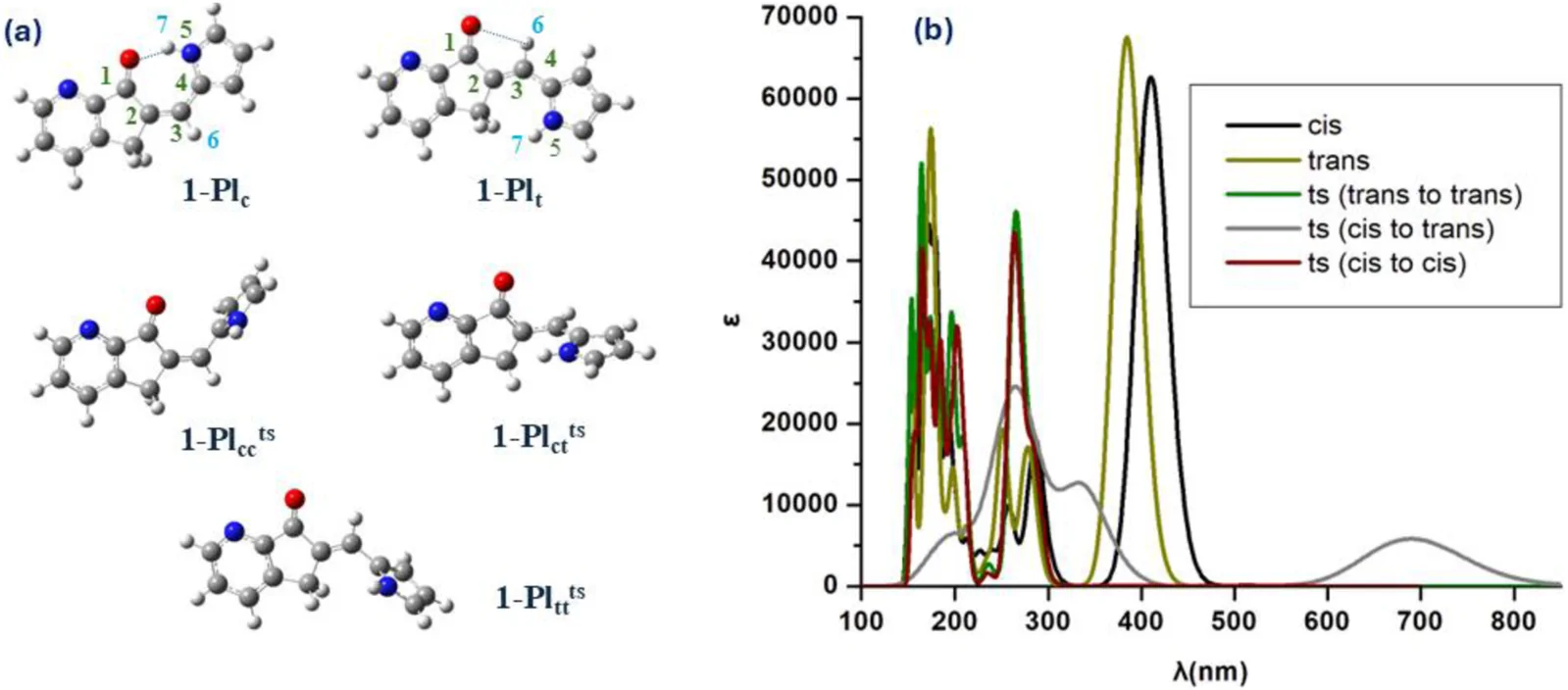
**(a)** Optimized *cis-, trans-* and transition states of **1-Pl (b)** absorption spectra of **1**-**Pl** at the PBE0/def2-TZVP level of theory in chloroform.

**FIGURE 7:**
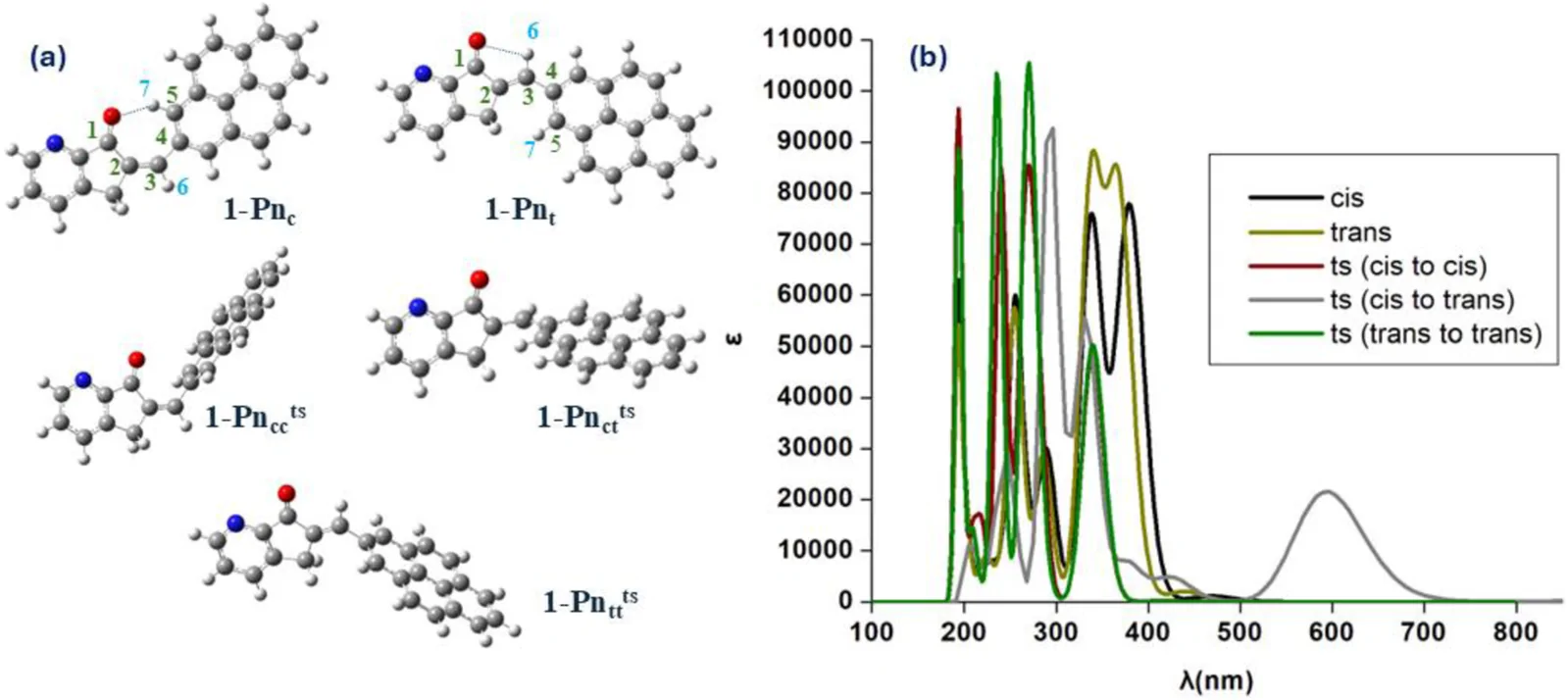
**(a)** Optimized *cis-, trans-* and transition states of **1-Pn (b)** absorption spectra of **1**-**Pn** at the PBE0/def2-TZVP level of theory in chloroform.

**TABLE 2:** O ^…^ H distances^a^ in Å, dihedral angle CCCC and CCCH values in degrees for the calculated structures at the S_0_ and S_1_ state.

Molecule	O ^…^ H^a^	CCCC^b^	CCCH^c^	O ^…^ H^a^	CCCC^b^	CCCH^c^
​	​	**S**_**0**_	​	​	**S**_**1**_	​
1-An_t_	2.475	30.54	1.68	2.410	3.26	0.36
1-An_c_	2.307	36.19	171.71	2.350	36.34	167.25
1-An_ct_^ts^	3.704	13.23	95.00	​	​	​
1-An_cc_^ts^	3.854	95.93	179.81	​	​	​
1-Na_t_	2.460	0.00	0.00	2.464	0.01	0.00
1-Na_c_	2.012	0.00	180.00	1.915	0.01	180.00
1-Na_ct_^ts^	3.278	2.23	98.38	​	​	​
1-Na_cc_^ts^	3.703	89.94	178.82	​	​	​
1-Na_tt_^ts^	2.595	87.80	0.15	​	​	​
1-Ph_t_	2.461	0.00	0.00	2.458	0.00	0.00
1-Ph_c_	1.967	0.00	180.00	2.220	31.17	165.42
1-Ph_ct_^ts^	2.625	13.60	104.48	​	​	​
1-Ph_cc_^ts^	3.709	91.59	179.15	​	​	​
1-Pl_t_	2.530	0.02	0.01	2.487	0.02	0.00
1-Pl_c_	1.723	0.00	179.99	1.578	0.00	180.00
1-Pl_ct_^ts^	3.566	1.94	83.76	​	​	​
1-Pl_cc_^ts^	3.733	95.75	178.79	​	​	​
1-Pl_tt_^ts^	2.611	89.18	0.01	​	​	​
1-Pn_t_	2.461	0.00	0.00	2.505	180.00	0.00
1-Pn_c_	1.998	0.00	180.00	1.898	0.00	180.00
1-Pn_ct_^ts^	3.251	4.73	99.61	​	​	​
1-Pn_cc_^ts^	3.723	91.83	179.98	​	​	​
1-Pn_tt_^ts^	2.597	90.17	0.00	​	​	​

^a^ O ^…^ H6 for the 1-L_t_ and 1-L_tt_^ts^ structures and O ^…^ H7 for the 1-L_c_, 1-L_cc_^ts^ and 1-L_ct_^ts^ structures shown in Figures 3–7.

^b^ C2C3C4C5 as shown in Figures 3–7.

^c^ C1C2C3H6 as shown in Figures 3–7.

A van der Walls hydrogen bond is formed between the O atom of hemiindigo and the H atom of the L group in the case of the cis isomers or between the O atom and the H atom of the C=C bond in the case of the trans isomers. Specifically, in the case of the cis conformers, a seven-member ring is formed and the O ^…^ H7 distance ranges from 1.723 Å (**1-Pl**_**c**_) to 2.307 Å (**1-An**_**c**_), see Figures 3–7. Note that in the S_1_ excited state, the O ^…^ H7 distance ranges from 1.578 Å (**1-Pl**_**c**_) to 2.350 Å (**1-An**_**c**_), resulting to a decrease of the O ^…^ H7 distance in the case of anthracene and in an increase in the case of pyrrole. In the case of the trans conformers, a five-member ring is formed, see Figures 3–7. The O ^…^ H6 distance ranges from 2.460 Å (**1-Na**_**t**_) to 2.530 Å (**1-Pl**_**t**_). Note that in the S_1_ excited state, the O ^…^ H6 distance ranges from 2.410 Å (**1-An**_**t**_) to 2.505 Å (**1-Pn**_**t**_). Thus, the differences between the O ^…^ H distances of the S_0_ and S_1_ states range from 0.003 Å to 0.253 Å (**1-Ph**_**c**_), while the covalent bond distances are almost the same at both states.

All cis- and trans-conformations are planar, only for the 1-An_c_ isomer a small deviation from planarity is observed, i.e., the C1C2C3H6 dihedral angle is 171.7 degrees, while in S_1_ the deviation from planarity is increased to 167.3 degrees. Similarly, for the cis isomer at the S_1_ state the deviation from planarity is 165.4 degrees due to stereochemical factors.

The transition state of the isomerization reaction, **1-L**_**ct**_^**ts**^, has been calculated, where the plane of the hemiindigo is almost perpendicular to the plane of the = CHL. Specifically, the C1C2C3H6 dihedral angle, see Table 2, ranges from 83.3 degrees (**1-Pl**) to 104.5 degrees (**1-Ph**). The transition states of the isomerization reaction, **1-L**_**cc**_^**ts**^ and **1-L**_**tt**_^**ts**^, where the L group of the **1-L**_**c**_ or **1-L**_**t**_ is rotated, see Scheme 1, has been calculated; the C2C3C4C5 dihedral angle ranges from 83.3° (**1-Pl**) to 104.5 degrees (**1-Ph**).

Comparing the C2C3C4C5 and C1C2C3H6 dihedral angles of the S_0_ and S_1_ states, the largest differences are observed for **1-An**_**t**_ i.e., 27.3 degrees (C2C3C4C5) and for **1-Ph**_**c**_ i.e., -31.2 degrees (C2C3C4C5) 14.58 degrees C1C2C3H6). For the rest of the isomers of the molecules, the differences at these dihedral angles are up to 4.5 degrees.

### 3.3 Energetics and absorption spectra

Trans-structures present higher stability than cis-structures, with the exception of **1-Pl**. It exhibits a different behavior compared to the other molecules because the formed hydrogen bond O ^…^ HN is stronger that the O ^…^ HC bond. Thus, trans isomers are more stable than cis and their energy differences range from 0.147 eV to 0.184 eV, except for **1-Pl**, where the cis isomer is the most stable by −0.168 eV, see Table 3. The activation energy for the rotation of the L group of the **1-L**_**c**_ or **1-L**_**t**_, i.e., the ΔE (**1-L**_**cc**_^**ts**^ − **1-L**_**c**_) and ΔE (**1-L**_**tt**_^**ts**^ − **1-L**_**t**_) energies range from 0.077 eV (**1-Pn**_**cc**_^**ts**^) to 0.629 eV (**1-Pl**_**cc**_^**ts**^); the last one presents the highest activation energy due to the O ^…^ HN bond breaking, Table 3 and Figure 8.

**TABLE 3:** Energy differences ΔE (eV) of the trans, cis, and transition states of the **1-An**, **1-Na**, **1-Ph**, **1-Pl**, and **1-Pn** molecules at the PBE0/def2-TZVP level of theory in chloroform solvent.

Molecule	ΔE (1-L_c_ − 1-L_t_)	ΔE (1-L_tc_^ts^ − 1-L_t_)^a^	ΔE (1-L_cc_^ts^ − 1-L_c_)	ΔE (1-L_tt_^ts^ − 1-L_t_)
1-An	0.180	2.088 (594)	0.080	​
1-Na	0.184	2.100 (590)	0.082	0.228
1-Ph	0.147	2.021 (613)	0.250	​
1-PL	−0.168	2.013 (616)	0.629	0.429
1-Pn	0.176	2.108 (588)	0.077	0.204

^a^ Parentheses present the corresponding wavelengths (nm).

**FIGURE 8:**
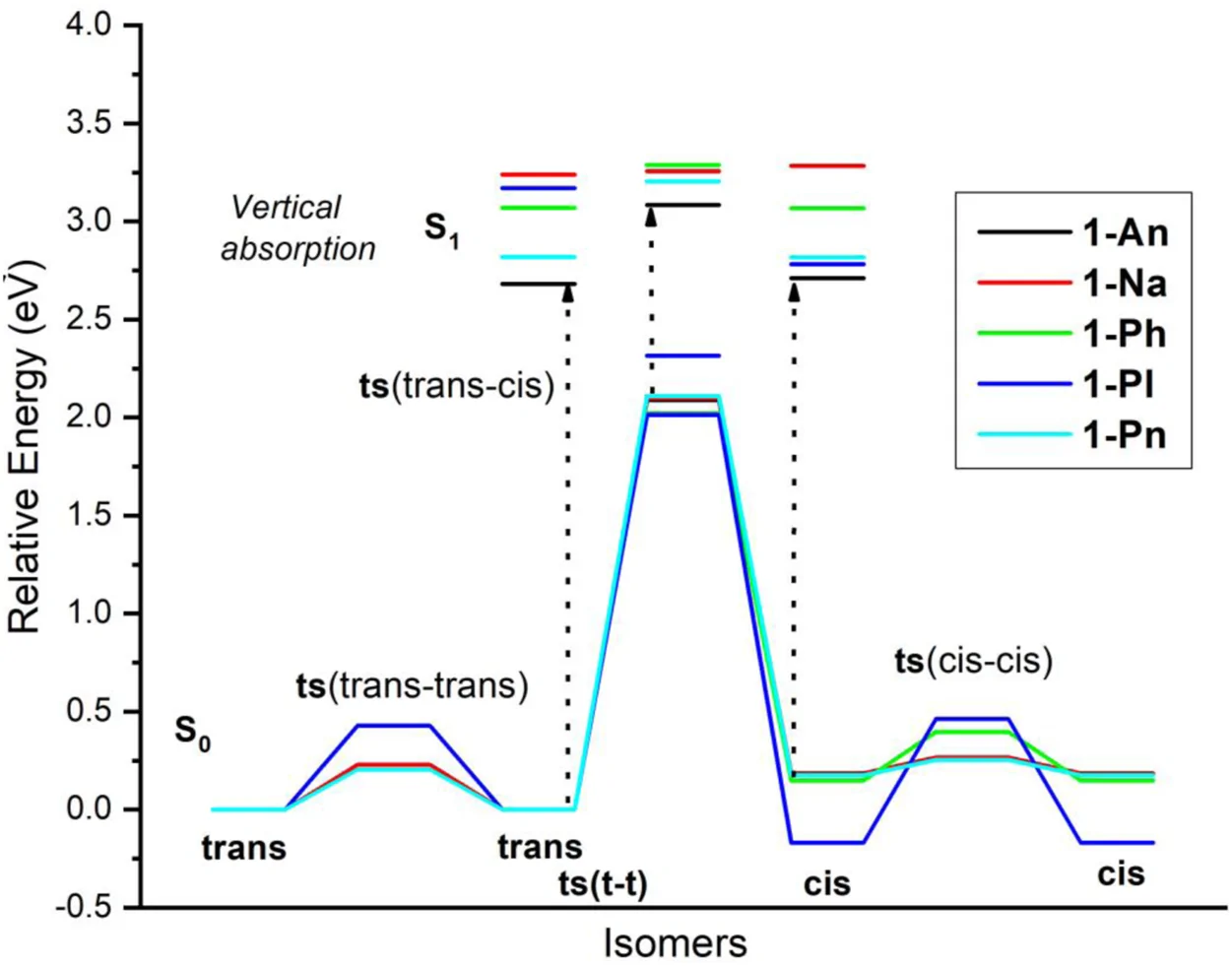
Relative energies of the trans, cis and transition states of the **1-An**, **1-Na**, **1-Ph**, **1-Pl**, and **1-Pn** molecules at the PBE0/def2-TZVP level of theory in chloroform solvent.

For the trans-cis isomerization, the **1-L**_**tc**_^**ts**^ transition state is about ∼2 eV higher in energy than the trans isomer, i.e., energy barrier ranges from 2.013 eV (**1-Pl**) to 2.108 eV (**1-Pn**), see Table 3. The corresponding λ values for these energy barriers fall within the visible region of the electromagnetic spectrum, suggesting that the cis–trans isomerization may be photoinduced by visible light. Energetically, after a vertical absorption, the S_1_ states of the trans and cis structures are close in energy, while the energy difference between the trans-cis transition at the ground state and the S_1_ state, after a vertical transition, i.e., vertical absorption, is about 1 eV, see Figure 8. After relaxation of the optimized trans and cis structures of the S_1_ state, the energy gap between the relaxed S_1_ and the transition state at the S_0_ is reduced and the energy gap ranges from 0.17 eV (**1-An**_**c**_) to 0.84 eV (**1-An**_**t**_).

Absorption spectra of the calculated minimum structures are shown in Figures 3–7. Also, main absorption peaks, energy differences of S→S, S→T, oscillator strength (*f*) values which express the probability of transitions between energy levels, and the corresponding main excitations of the absorption spectrum of the 1-L cis, trans and transition state species at PBE0/def2-TZVP are presented in Table 4.

**TABLE 4:** Absorption peaks, λ (nm), energy differences ΔE (eV) of S→S, S→T, f-values, and the corresponding main excitations of the absorption spectrum of the **1-L** cis, trans and transition state species at the PBE0/def2-TZVP level of theory in chloroform solvent.

Molecule	State	λ	ΔE	f	Main excitation
1-An_t_	S_1_	462.3	2.68	0.305	0.995 |H → L>
​	S_5_	323.6	3.83	0.339	0.713 |H-1 → L>
​	S_14_	249.6	4.97	1.517	0.666 |H-2 → L+1>
1-An_c_	S_1_	490.3	2.53	0.287	0.975 |H → L>
​	S_5_	324.7	3.82	0.278	0.711 |H-3 → L>
​	S_13_	256.8	4.83	0.277	0.905 |H-6 → L>
​	S_14_	250.4	4.95	1.503	0.584 |H → L+3> + 0.567 |H-2 → L+1>
1-An_cc_^ts^	S_1_	497.9	2.49	0.001	0.971 |H → L>
​	S_11_	268.7	4.61	0.649	0.801 |H-5 → L>
​	S_15_	249.1	4.98	1.271	0.484 |H-1 → L+1> + 0.460 |H → L+3>
1-An_ct_^ts^	S_1_	1,245	1.00	0.151	0.993|H → L>
​	S_2_	662.0	1.87	0.211	0.896|H-1 → L>
​	S_27_	239.8	5.17	0.508	0.599 |H-11 → L>
1-Na_t_	S_1_	383.3	3.23	0.659	0.987 |H → L>
​	S_13_	238.5	5.20	0.600	0.571 |H-1 → L+1>
1-Na_c_	S_1_	399.8	3.10	0.497	0.983 |H → L>
​	S_3_	351.7	3.53	0.507	0.971 |H-1 → L>
​	S_13_	241.4	5.14	0.463	0.585 |H-1 → L+1>
1-Na_cc_^ts^	S_1_	429.1	2.89	0.001	0.703 |H → L>
​	S_8_	266.5	4.65	0.639	0.913 |H-4 → L>
​	S_17_	218.1	5.68	1.184	0.567 |H → L+3>
1-Na_ct_^ts^	S_1_	1,071	1.16	0.134	0.982 |H → L>
​	S_2_	609.3	2.03	0.190	0.943 |H-1 → L>
​	S_18_	246.4	5.03	0.867	0.863 |H-2 → L+1>
1-Na_tt_^ts^	S_1_	396.7	3.13	0.001	0.885 |H → L>
​	S_8_	268.2	4.62	0.714	0.906 |H-4 → L>
​	S_17_	218.1	5.68	1.184	0.567 |H → L+3>
1-Ph_t_	S_1_	403.3	3.07	0.689	0.993 |H → L>
​	S_10_	257.7	4.81	0.422	0.712 |H-1 → L+1>
1-Ph_c_	S_1_	424.6	2.92	0.580	0.993 |H → L>
1-Ph_cc_^ts^	S_1_	2074	0.60	0.036	1.000 |H → L>
​	S_2_	669.4	1.85	0.148	0.964 |H-1 → L>
​	S_4_	458.5	2.70	0.166	0.747 |H-3 → L>
​	S_26_	238.9	5.19	0.411	0.803 |H-2 → L+2>
1-Ph_ct_^ts^	S_1_	979.3	1.27	0.134	0.974 |H → L>
​	S_2_	579.3	2.14	0.171	0.934 |H-1 → L>
1-Pl_t_	**S**_**1**_	391.7	3.17	0.753	1.000 |H → L>
​	S_25_	174.8	7.09	0.374	0.714 |H-6 → L+1>
1-Pl_c_	**S**_**1**_	419.9	2.95	0.697	0.999 |H → L>
1-Pl_cc_^ts^	S_2_	688.3	1.80	0.071	0.995 |H-1 → L>
​	S_5_	348.6	3.56	0.200	0.937 |H → L+1>
1-Pl_ct_^ts^	S_2_	688.2	1.80	0.071	0.995 |H-1 → L>
​	S_11_	270.0	4.58	0.468	0.670 |H-6 → L> + 0.654 |H-5 → L>
1-Pl_tt_^ts^	S_1_	453.0	2.74	0.001	0.956 |H → L>
​	S_7_	265.1	4.68	0.471	0.917 |H-4 → L>
1-Pn_t_	S_1_	439.8	2.82	0.023	0.978 |H → L>
​	S_3_	367.9	3.37	0.875	0.981 |H-1 → L>
​	S_4_	338.1	3.67	0.900	0.946 |H → L+1>
1-Pn_c_	S_1_	468.9	2.64	0.012	0.986 |H → L>
​	S_3_	379.7	3.27	0.860	0.987 |H-1 → L>
​	S_4_	338.4	3.66	0.836	0.947 |H → L+1>
1-Pn_cc_^ts^	S_1_	445.1	2.79	0.001	0.995 |H → L>
​	S_12_	266.5	4.65	0.720	0.893 |H-5 → L>
​	S_19_	239.6	5.17	0.745	0.808 |H-1 → L+2>
1-Pn_ct_^ts^	S_1_	1,130	1.10	0.125	0.988 |H → L>
​	S_2_	621.0	2.00	0.028	0.988 |H-1 → L>
​	S_3_	591.8	2.10	0.219	0.955 |H-2 → L>
​	S_9_	333.0	3.72	0.553	0.664 |H-1 → L+1> + 0.692 |H → L+2>
1-Pn_tt_^ts^	S_1_	418.4	2.96	0.002	0.996 |H → L>
​	S_12_	267.9	4.63	0.633	0.778 |H-5 → L>
​	S_20_	235.7	5.26	1.098	0.913 |H-1 → L+2>

The absorption spectrum of the five molecules presents the same general shape. The main vis peak of the trans and cis isomers is observed in the region between 383 nm and 490 nm. The main vis peak of cis isomers is red shifted with respect to the trans one on average by about 25 nm. The transition state of the trans-cis isomerization presents a main vis peak in the region between 579 nm and 688 nm. Moreover, the transition state of the trans-trans or cis-cis isomerization, i.e., **1-L**_**cc**_^**ts**^ and **1-L**_**tt**_^**ts**^, where the L group of the **1-L**_**c**_ or **1-L**_**t**_ is rotated, presents a vis red shifted main peak by 10 nm with respect to the corresponding cis or trans isomers, respectively. Finally, in all cases the peaks of higher intensity are found in the UV region rather than in the visible part of the electromagnetic spectrum.

In the case of **1-An**, the main vis peak of the **1-An**_**t**_ conformer corresponds to a HOMO (H) → LUMO (L) transition and it is calculated at 462 nm, in good agreement with the experimental value of 446 nm (Quintana-Romero and Ariza-Castolo, 2021). Note that it is red shifted by 16 nm (only 0.10 eV) and shifts up to 0.2 eV are considered as showing very good agreement between experimental and TDDFT calculations (Aprahamian, 2020). For the cis isomer, the vis main peak is red shifted, while both cis and trans isomers have vis main peaks with similar f-values. On the contrary, the **1-An**_**ct**_^**ts**^ presents a main vis peak at 662 nm (1.87 eV), i.e., significantly red shifted, by ∼200 nm with respect to the cis and trans isomers, see Table 4. Finally, the main UV peak for all calculated structures, **1-An**_**t**,_ **1-An**_**c**,_ **1-An**_**cc**_^**ts**^ and **1-An**_**ct**_^**ts**^ is at ∼250 nm, and **1-An**_**t**_ presents the most intense peak, i.e., the largest f-value.

Regarding **1**-**Na**, the highest peak for trans-conformer is at 383 nm, while for cis is at 400 nm, i.e., they both absorb in the *violet* area of the visible spectrum. The peak corresponds to a H → L transition with an f-value of 0.66 and 0.50 respectively. **1-Na**_**ct**_^**ts**^ presents a main vis peak at 609 nm (2.03 eV), i.e., it is red shifted by about ∼200 nm as in the case of **1-An**. The main UV peak for all calculated **1-Na** structures (**1-Na**_**t**,_ **1-Na**_**c**,_ **1-Na**_**cc**_^**ts**^, **1-An**_**tt**_^**ts**^ and **1-An**_**ct**_^**ts**^) is observed at ∼240 nm and at ∼218 nm, where the second one is the strongest one with an f-value of about 1.18. Note that, contrary to the trans and cis isomers, the H → L transition for the **1-Na**_**cc**_^**ts**^ and **1-An**_**tt**_^**ts**^ structures has a charge transfer character, and the f-value is very small, see Table 4 and discussion below.

For **1-Ph**, the first absorption peak of the trans-conformer is at 403.3 nm, while for cis is at 425 nm; they correspond to a H → L transition with an f-value of 0.69 and 0.59 respectively. Both **1-Ph**_**ct**_^**ts**^ and **1-Ph**_**cc**_^**ts**^ present a main vis absorption peak at 579 nm and 669 nm, respectively, which corresponds to S_0_ → S_2_ excitation, i.e., a H-1 → L transition. The S_0_ → S_1_ excitation, which corresponds to a H → L transition, is observed at the NIR region and only for the **1-Ph**_**ct**_^**ts**^ structure, the peak is found at 979 nm with an f-value of the similar intensity with the main vis peak, i.e., f = 0.13, while for the **1-Ph**_**cc**_^**ts**^ the f-value is very small, i.e., 0.036, see Table 4. Finally, the UV peak of the isomers of **1-Ph** is found at ∼260 nm.

Following, in **1**-**Pl**, the first main peak of **1-Pl**_**t**_ results from the S_0_ → S_1_ excitation and corresponds to a H → L transition. It is calculated at 392 nm, i.e., in very good agreement with the experimental value of 400 nm (Quintana-Romero and Ariza-Castolo, 2021). The corresponding **1**-Pl_c_ is at 420 nm. Both isomers present intense main vis peaks, see Figure 6 and Table 4. Both **1-Pl**_**ct**_^**ts**^ and **1-Pl**_**cc**_^**ts**^ present a main vis absorption peak at 668 nm, and corresponds to S_0_ → S_2_ excitation, i.e., to a H-1 → L transition. The three calculated transition states, **1**-**Pl**_**cc**_^**ts**^, **1**-Pl_ct_^ts^, and **1**-Pl_tt_^ts^ present main peaks at ∼270 nm, see Figure 6.

Last, in **1-Pn**, the first absorption peak for **1**-Pn_t_ is at 440 nm, while for **1**-**Pn**_**c**_ is at 468.9 nm, see Figure 7. Both peaks are not intense, i.e., their f-value is about 0.02. Their main UV-vis peaks are at 368 nm for **1**-Pn_t_ and 380 nm for **1**-Pn_c_ indicating an approximate difference of about 30 nm, while the oscillator strength is about 0.9, see Table 4. The main UV peaks are observed at 338 nm. Finally, **1-Pn**_**ct**_^**ts**^ presents a main vis absorption peak at 621 nm that corresponds to S_0_ → S_2_ excitation, i.e., a H-1 → L transition. On the contrary, the transition states, **1**-**Pn**_**cc**_^**ts**^, and **1-Pl**_**tt**_^**ts**^, present main peaks at 445 and 418 nm, respectively, see Figure 7.

The electron density distribution in frontiers molecular orbitals for all molecules are shown in Figure 9 and Supplementary Figures S9-S13 of the SI. Pyrrole has a N atom and is a π-excessive aromatic system. Naphthalene has two fused benzene rings. Anthracene and phenanthrene have three fused benzene rings, but the latter is more stable, while pyrene has four fused benzene rings and thus the electron density can be delocalized in a larger fused system. For all molecules, the excitations of interest are S_0_ → S_1_, S_0_ → S_2_ and S_0_ → S_3_. Charge transfer (CT), where a partial charge is transferred, and electron transfer (ET), where an electron is transferred from one group of the molecules to another group are observed.

**FIGURE 9:**
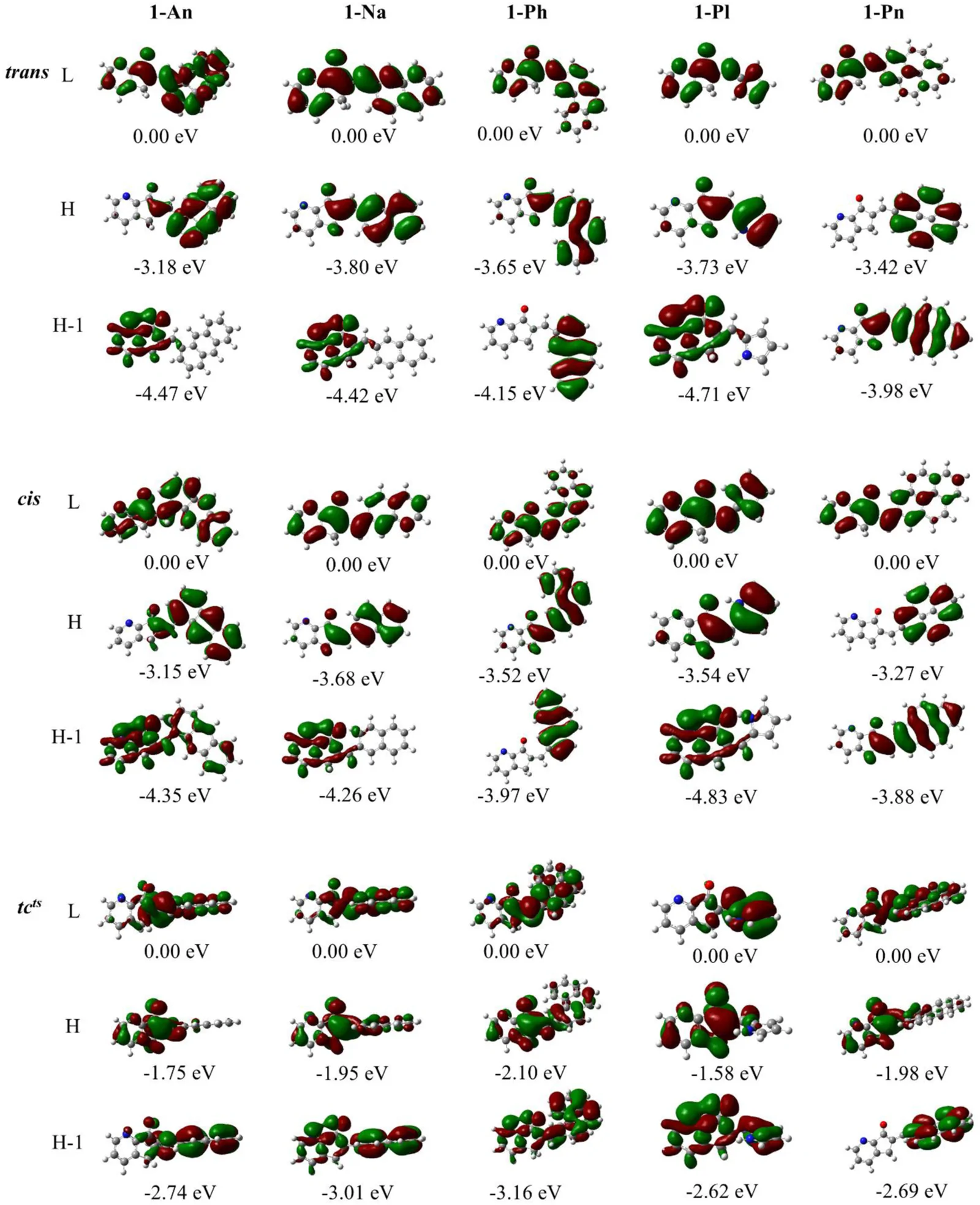
Frontier Molecular Orbitals of the **1-An**, **1-Na**, **1-Ph**, **1-Pl**, and **1-Pn** molecules at the PBE0/def2-TZVP level of theory in chloroform solvent.

Comparing the frontiers H-1, H and L orbitals of the trans, cis and their transition state, it is found that these orbitals are the same for both trans and cis isomers, while they differ from orbitals of the transition state, i.e., their electron density is in different groups, see Figure 9.

The electron density of the L orbital of the trans and cis isomers of the **1-An**, **1-Na**, **1-Ph**, and **1-Pl** molecules is delocalized in the whole molecule, while at **1-Pn**, which has the most extended π-system, the electron density of the L orbital is mainly located to the hemiindigo. On the contrary, the electron density of the H orbitals in the case of the **1-An**, **1-Na**, **1-Ph**, and **1-Pl** molecules is localized in the L group and at the 5-member ring of the hemiindigo, while at **1-Pn**, which has the most extended π-system, the electron density of the L orbital is only located at the L = pyrene group. Finally, the electron density of the H-1 orbitals in the case of the **1-An**, **1-Na**, and **1-Pl** molecules is localized at the hemiindigo group, in the case of **1-Ph** the electron density is localized at the L = phenanthrene group, while in the case of **1-Pn** molecule is localized in the L group and at the 5-member ring of the hemiindigo. Thus, the difference in the localization of the electron density of the H-1, H, and L results in charge transfer or electron transfer low-lying electronic excitations.

For both cis and trans isomers, the S_0_ → S_1_ excitation corresponds to a H → L transition, which is a charge transfer process from the L group and the 5-ring member ring of the hemiindigo to the whole molecule for the **1-An**, **1-Na**, **1-Ph**, and **1-Pl** molecules. On the contrary, for **1-Pn**, the S_0_ → S_1_ excitation is an electron transfer excitation from the pyrene group to hemiindigo. Finaly, in the H-1 → L, H → L+1, and H-1 → L+1 transitions, the electron density is delocalized over the whole molecule, either from the hemiindigo or the L group.

### 3.4 Emission spectra

The emission spectra of the trans and cis isomers of the **1-An**, **1-Na**, **1-Ph**, **1-Pl**, and **1-Pn** molecules have been plotted in Figure 10, and the emission peaks are given in Table 5. All are located in the vis region. The molecules emit at different λ values, covering all the regions of the visible spectrum, Figure 10. The λ values of the calculated molecules range from 432 nm to 792 nm. Specifically, the λ values of the trans isomers range from 432 nm to 558 nm, while the λ values of the cis isomers range from 446 nm to 792 nm, see Table 5.

**FIGURE 10:**
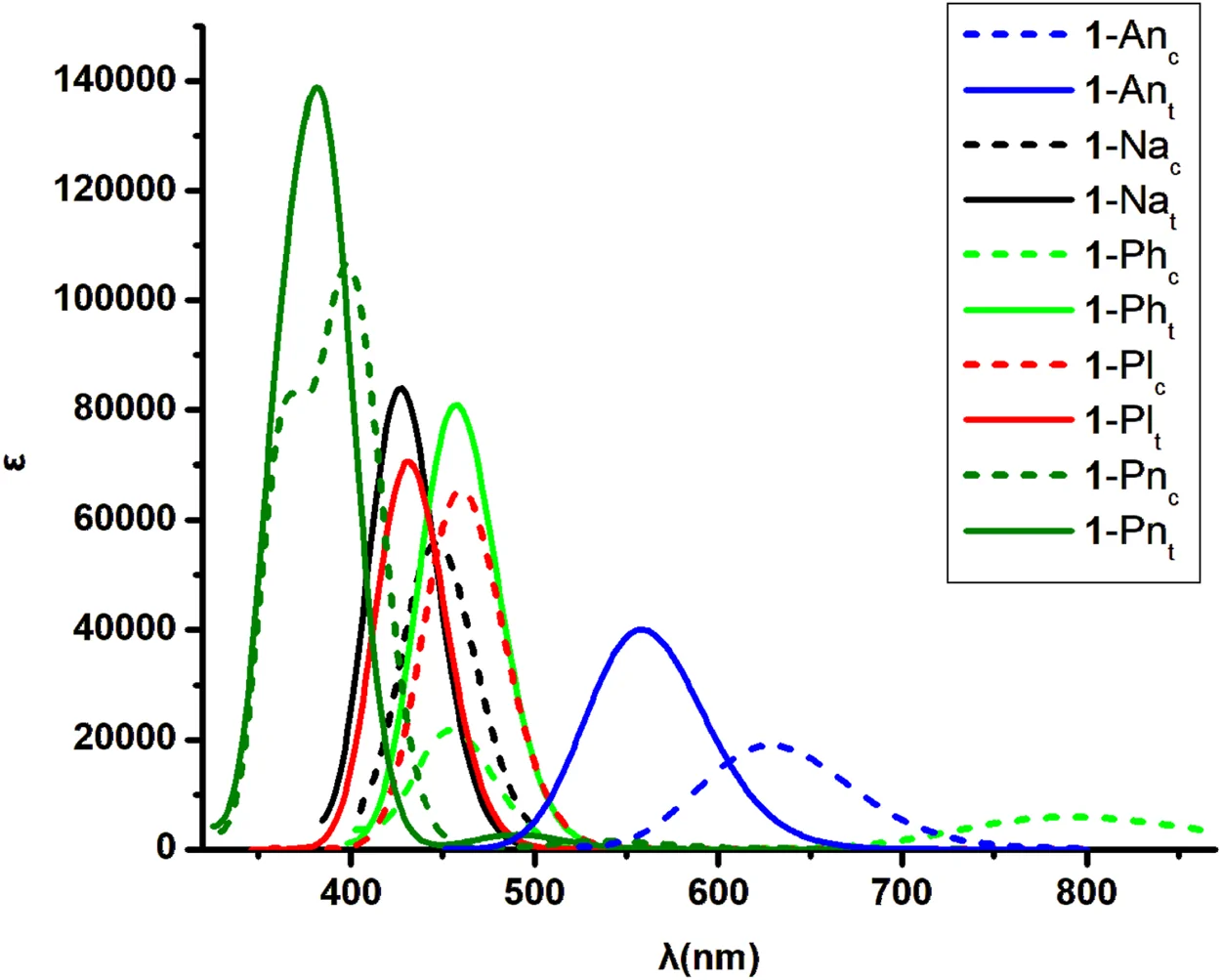
Emission spectra for the cis and trans isomers of the **1-An, 1-Na, 1-Ph, 1-Pn,** and **1-Pl** molecules at the PBE0/def2-TZVP method in chloroform solvent.

**TABLE 5:** Emission peaks, λ (nm), energy differences ΔE (eV) of S_x_ → S_o_ de-excitations, f-values, and the corresponding main excitations of the emission spectrum of the 1-L cis and trans species at the PBE0/def2-TZVP level of theory in chloroform solvent.

Molecule	De-excitation	λ	ΔE	f	Main excitation
1-An_t_	S_1_ → S_0_	558.4	2.22	0.446	0.997 |H → L>
1-An_c_	S_1_ → S_0_	628.4	1.97	0.211	0.955 |H → L>
1-Na_t_	S_1_ → S_0_	427.2	2.90	0.934	0.993 |H → L>
1-Na_c_	S_1_ → S_0_	446.6	2.78	0.622	0.992 |H → L>
1-Ph_t_	S_1_ → S_0_	457.4	2.71	0.900	0.995 |H → L>
1-Ph_c_	S_1_ → S_0_	791.9	1.57	0.065	0.967 |H → L>
​	S_2_ → S_0_	450.9	2.75	0.261	0.959 |H-1 → L>
1-Pl_t_	S_1_ → S_0_	431.5	2.87	0.785	0.998 |H → L>
1-Pl_c_	S_1_ → S_0_	460.0	2.70	0.731	1.000 |H → L>
1-Pn_t_	S_1_ → S_0_	490.3	2.53	0.029	0.987 |H → L>
​	S_2_ → S_0_	387.2	3.20	1.293	0.991 |H-1 → L>
1-Pn_c_	S_1_ → S_0_	537.3	2.31	0.016	0.992 |H → L>
​	S_2_ → S_0_	400.2	3.10	1.135	0.992 |H-1 → L>

It is interesting that the fluorescence spectrum of the cis isomer is significantly red shifted with respect to the spectrum of trans isomer, contrary to the absorption spectra where the differences range from 10 nm to 30 nm. The calculated difference between the λ values of the trans and cis isomers are found at: 70 nm for **1-An**, 19 nm for **1-Na**, 335 nm for **1-Ph**, 29 nm for **1-Pl**, and 47 nm for **1-Pn**. Note that the significant red shift of the fluorescence peak of the cis isomer with respect to the trans isomer of the **1-Ph** molecule exists with a simultaneous significant reduction of the intensity of the peak. Overall, for all molecules, the intensity of the fluorescence spectra of the cis isomers is smaller than the intensity of the trans spectra.

The vertical absorption, vertical emission and adiabatic energies differences are given in Table 6. The vertical absorption energy corresponds to the energy required for the S_0_ → S_1_ excitation at the optimized ground-state geometry. Since electronic excitation occurs much faster than nuclear motion, the molecule initially reaches the S_1_ state, while retaining the S_0_ geometry. It then undergoes structural and vibrational relaxation to the optimized S_1_ geometry. From this relaxed S_1_ state, the molecule can return radiatively to S_0_ by emitting a photon; this is the S_0_ → S_1_ fluorescence transition, i.e., the vertical emission energy. Finally, the molecule relaxes at the optimized S_0_ geometry. The energy difference between the energies of the geometry optimized S_0_ and S_1_ states corresponds to the adiabatic energy differences. For all molecules, with the exception of **1-Ph**_**c**_, the order of energy differences is ΔE _(abs-vert)_ > ΔE _(adiab)_ > ΔE _(em-vert),_ while for **1-Ph**_**c**_ the order is reversed ΔE _(em-vert)_ > ΔE _(adiab)_ > ΔE _(abs-vert)_. Differences among these three values show differences in the relaxed geometries at the S_0_ and S_1_ states. The largest energy difference between the vertical absorption and vertical emission energies are observed for **1-An**_**c**_ at 0.56 eV and for **1-Ph**_**c**_ at 0.53 eV. Finally, the energy demands for the vertical absorption energy range from 2.53 eV to 3.24 eV, the energy gain for the vertical emission energy ranges from 1.97 eV to 3.45 eV, while the adiabatic energy difference ranges from 2.26 eV (**1-An**_**c**_) to 3.07 eV (**1-Ph**_**c**_). The variety in energy difference (Table 6; Figure 10) in conjunction with the fact that irradiation can induce photoisomerization around double bonds (Figure 8) can make them potential candidates for photoswitches and molecular logic gates.

**TABLE 6:** Vertical absorption, vertical emission and adiabatic energy differences (in eV) for the trans and isomer of the **1-An, 1-Na, 1-Ph, 1-Pn**, and **1-Pl** molecules at PBE0/def2-TZVP in chloroform solvent.

Molecule	ΔE _(abs-vert)_	ΔE _(em-vert)_	ΔE _(adiab)_
1-An_t_	2.68	2.22	2.36
1-An_c_	2.53	1.97	2.26
1-Na_t_	3.24	2.90	3.04
1-Na_c_	3.10	2.78	2.91
1-Ph_t_	3.07	2.71	2.86
1-Ph_c_	2.92	3.45	3.07
1-Pl_t_	3.17	2.87	3.01
1-Pl_c_	2.95	2.70	2.81
1-Pn_t_	2.82	2.53	2.67
1-Pn_c_	2.64	2.31	2.47

### 3.5 Application as MLGs

A photoswitch is a molecule that alters its geometry and chemical properties when exposed to electromagnetic radiation. Irradiation can induce photoisomerization around double bonds, converting between cis and trans configurations. In general, photochromic compounds are essential components for building light-driven molecular motors and machines. Thus, these photoresponsive molecules are being explored for a variety of applications (Aprahamian, 2020; Kassem et al., 2017), such as fluorescence probe for sensing (Bhakta et al., 2024), theranostic tools (Miya et al., 2026), photoswitches (Ghosh et al., 2024), biocompatible viscosity probes (Samanta et al., 2024) etc. Specifically, hemiindigoid photoswitches offer a flexible and promising foundation for innovation in many areas. Advancing their applications will require continued interdisciplinary work that brings together synthetic chemistry, materials science, and biology.

Irradiation of the molecules with light of 580–620 nm can induce photoisomerization around double bonds converting between cis and trans configurations depending on the L group. It is interesting to investigate if these specific molecules have the potential to be used as molecular logic gates. Thus, considering the irradiation with light of specific λ values and the absorption of light with specific λ values as ‘inputs”, while the emission at specific λ values as “output”, it is found that depending on the λ values, the 1-L can be used as “AND” and “Converse nonimplication” molecular logic gates. Table 7 is the corresponding truth table that shows that these molecules can be used as MLGs. A threshold f-value can be used for the discrimination between the ‘‘on’’ states, i.e., output = 1 and ‘‘off’’ states, i.e., output = 0. Here, a f-value that is 1/5 of the main peak is used as an output = 0. Note that the threshold of one-fifth of the main absorption peak was selected as a practical criterion to distinguish the high-intensity (“on”) and low-intensity (“off”) states. It provides a clear separation between the pronounced absorption associated with the “on” state and the weak residual absorption assigned to the “off” state. We agree that the robustness of the predicted logic operation with respect to the selected threshold is important. However, inspection of the calculated spectra indicates that moderate changes in the threshold would not alter the qualitative “on/off” assignments or the resulting logic operation.

**TABLE 7:** Truth tables resulting from 1-L using photoisomerization and absorption as input and emission modes as output.

Input	Input	Output	Output	Input	Input	Output
1-An_t_				1-Pn_t_		
Irradiation 594 nm	Absorption 480 nm	Emission 558 nm	Emission 628 nm	Irradiation 588 nm	Absorption 440 nm	Emission 537 nm
0	0	0	0	0	0	0
0	1	1	0	0	1	0
1	0	0	0	1	0	0
1	1	0	1	1	1	1
MLG	​	Converse nonimplication	AND	​	​	AND

1-Na_t_				1-Ph_t_		
Irradiation 590 nm	Absorption 380 nm	Emission 420 nm	Emission 446 nm	Irradiation 613 nm	Absorption 400 nm	Emission 780 nm
0	0	0	0	0	0	0
0	1	1	0	0	1	0
1	0	0	0	1	0	0
1	1	0	1	1	1	1
MLG	​	Converse nonimplication	AND	​	​	AND

1-Pl_c_						
Irradiation 616 nm	Absorption 400 nm	Emission 420 nm	Emission 477 nm	​	​	​
0	0	0	0	​	​	​
0	1	0	1	​	​	​
1	0	0	0	​	​	​
1	1	1	0	​	​	​
MLG	​	AND	Converse nonimplication	​	​	​

Specifically, for **1-An**_**t**_, subsequent irradiation at 594 nm and 480 nm, and emission output at 558 nm and 628 nm can lead **1-An**_**t**_ to be “Converse nonimplication” and “AND” MLG, respectively. Similarly, subsequent irradiation at two different λ values, can lead **1-Pn**_**t**_ to be AND MLG at 537 nm, **1-Ph**_**t**_ to be AND MLG at 780 nm, **1-Na**_**t**_ to be “Converse nonimplication” MLG at 420 and “AND” MLG at 446 nm MLG, and **1-Pl**_**c**_ to be “AND” MLG at 420 nm MLG and “Converse nonimplication” MLG at 477 nm. Finally, it should be noted that small differences can exist between the experimental and computational spectra, so the experimental λ values may be shifted. Nevertheless, even accounting for these shifts, the present theoretical study taking into consideration the calculated absorption and emission spectra, show that theoretically these hemiindigo derivatives have the potential to be used as molecular logic gates and photoswitches. Thus, the present study evaluates the feasibility of these processes but does not determine experimental quantum yields or switching efficiencies; instead, it provides a theoretical basis for further experimental investigation.

## 4 Summary and conclusion

Hemiindigoid photoswitches offer a flexible and promising foundation for innovation in many areas. Here, the photophysical properties of a series of pseudo-hemiindigo derivatives with anthracene, naphthalene, phenanthrene, pyrene, and pyrrole, i.e., **1-L**, L = anthracene (An), phenanthrene (Ph), naphthalene (Na), pyrenene (Pn), and pyrrole (Pl), are studied via DFT (PBE0, B3LYP, M06–2X, ωB97XD and CAM-B3LYP) calculations.

Trans-structures present higher stability than cis-structures, with the exception of **1-Pl**. It exhibits a different behavior compared to the other molecules because the formed hydrogen bond O ^…^ HN is stronger that the O ^…^ HC bond.

The cis-trans isomerization as well as the cis-cis and trans-trans isomerization processes are investigated as well as their effect on the absorption and fluorescence spectra of the molecules. The isomerization process affects the absorption and fluorescence spectra, i.e., shifts of up to 30 nm and 70 nm are observed, respectively, while the transition state is shifted up to about 150 nm. Irradiation with light of 580–620 nm can induce photoisomerization around double bonds converting between cis and trans configurations depending on the L group.

The energy demands for the vertical absorption energy of the molecules range from 2.53 eV to 3.24 eV, the energy gain for the vertical emission energy ranges from 1.97 eV to 3.45 eV, while the adiabatic energy difference ranges from 2.26 eV to 3.07 eV. The variety in energy difference in conjunction with the fact that irradiation can induce photoisomerization around double bonds can make them potential candidates for photoswitches and molecular logic gates. However, it should be noted that the calculated absorption energies and isomerization barriers explain whether such processes are feasible and cannot be directly related to experimental photochemical quantum yields or switching efficiencies, as experimental validation is required for this purpose.

It is found that all have the potential to be used as two-input molecular logic gates, where two subsequent irradiations are used as inputs. Emission outputs at about 420, 450, 480, 540, 560, 630, 780 nm can be utilized as output to build AND and “Converse Nonimplication” logic gates. Finally, it should be noted that small differences between the experimental and computational spectra can lead to shifts in the experimental λ values that are used as inputs or outputs. But, even so, the present study demonstrates that these hemiindigo derivatives have the potential to be used as molecular logic gates (MLG) and photoswitches.

Although the results presented in this study demonstrate the potential application of the investigated systems as MLGs, several practical aspects should be considered when translating these computational findings into experimental applications. Specifically, selective detection of the output signal and sufficiently large spectral or intensity differences between the corresponding “on” and “off” states are necessary for the reliable implementation of the proposed logic operations. Another important consideration is photostability, because repeated irradiation can lead to photodegradation and thus affect the reproducibility of the switching process. Additionally, the lifetime and stability of the photoinduced states can be affected by thermal back-isomerization, while background fluorescence may interfere with the detection of the desired emission signals, particularly when the difference between the “on” and “off” states is relatively small. These factors would require experimental investigation, however the calculated photophysical properties and logical responses furnish a useful theoretical basis for the assessment of the potential use of these systems as photoswitches and MLGs.

## Data Availability

The original contributions presented in the study are included in the article/Supplementary Material, further inquiries can be directed to the corresponding author.
