# Supplementary material for: From photoisomerization to logic operations: a theoretical study of pseudo-hemiindigo derivatives
Source: Front Chem. 2026 Oct 14;14:1962171. doi: 10.3389/fchem.2026.1962171 (PMC13713479; doi:10.3389/fchem.2026.1962171)
Supplement: Supplementary file 1 [file DataSheet1.pdf]

Supporting Information

From Photoisomerization to Logic Operations: A Theoretical Study of Pseudo-Hemiindigo Derivatives

Christina Eleftheria Tzeliou [1], Demeter Tzeli [1, 2*]

[1] *Laboratory of Physical Chemistry, Department of Chemistry, National and Kapodistrian University of Athens, Panepistimiopolis Zografou, 157 84 Athens, Greece*

[2] *Theoretical and Physical Chemistry Institute, National Hellenic Research Foundation, 48 Vassileos Constantinou Ave., 116 35 Athens, Greece*

*Correspondence: tzeli@chem.uoa.gr

Tel.: +30-2107274307

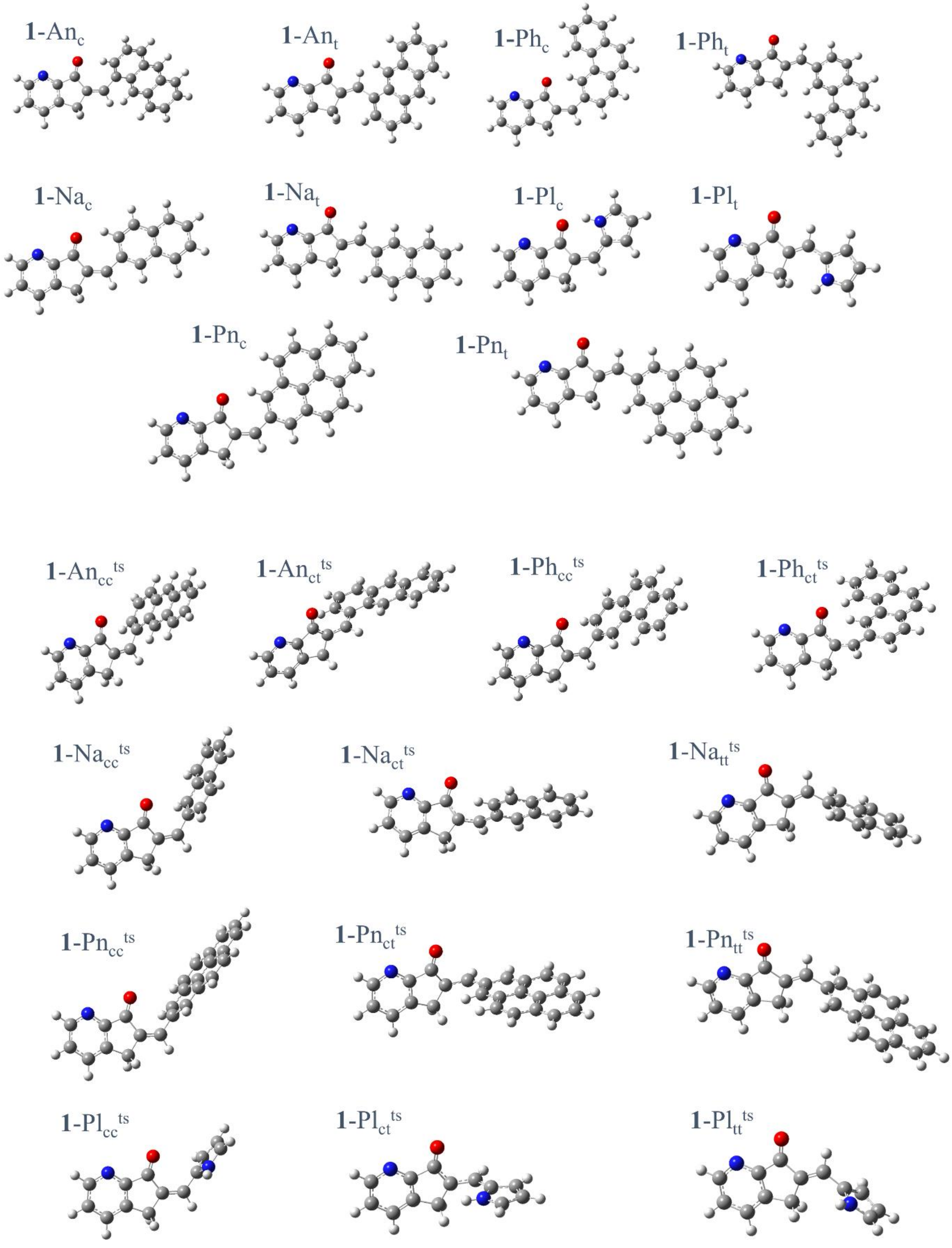

Figure S1: Optimized *cis*-, *trans*- structures and transition states of the molecules under study.

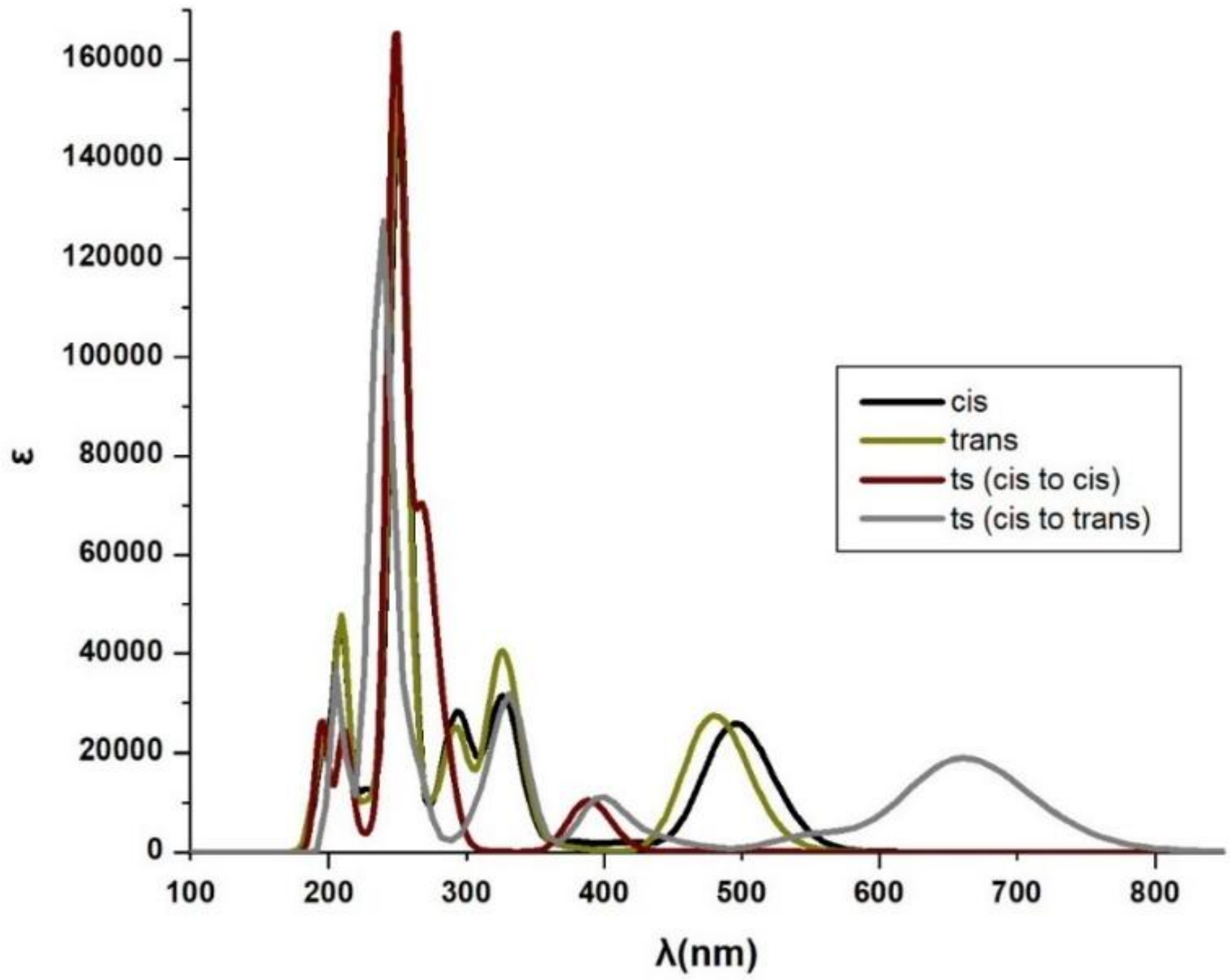

Figure S2: Absorption spectra of **1**-An at the PBE0/def2-TZVP level of theory in chloroform.

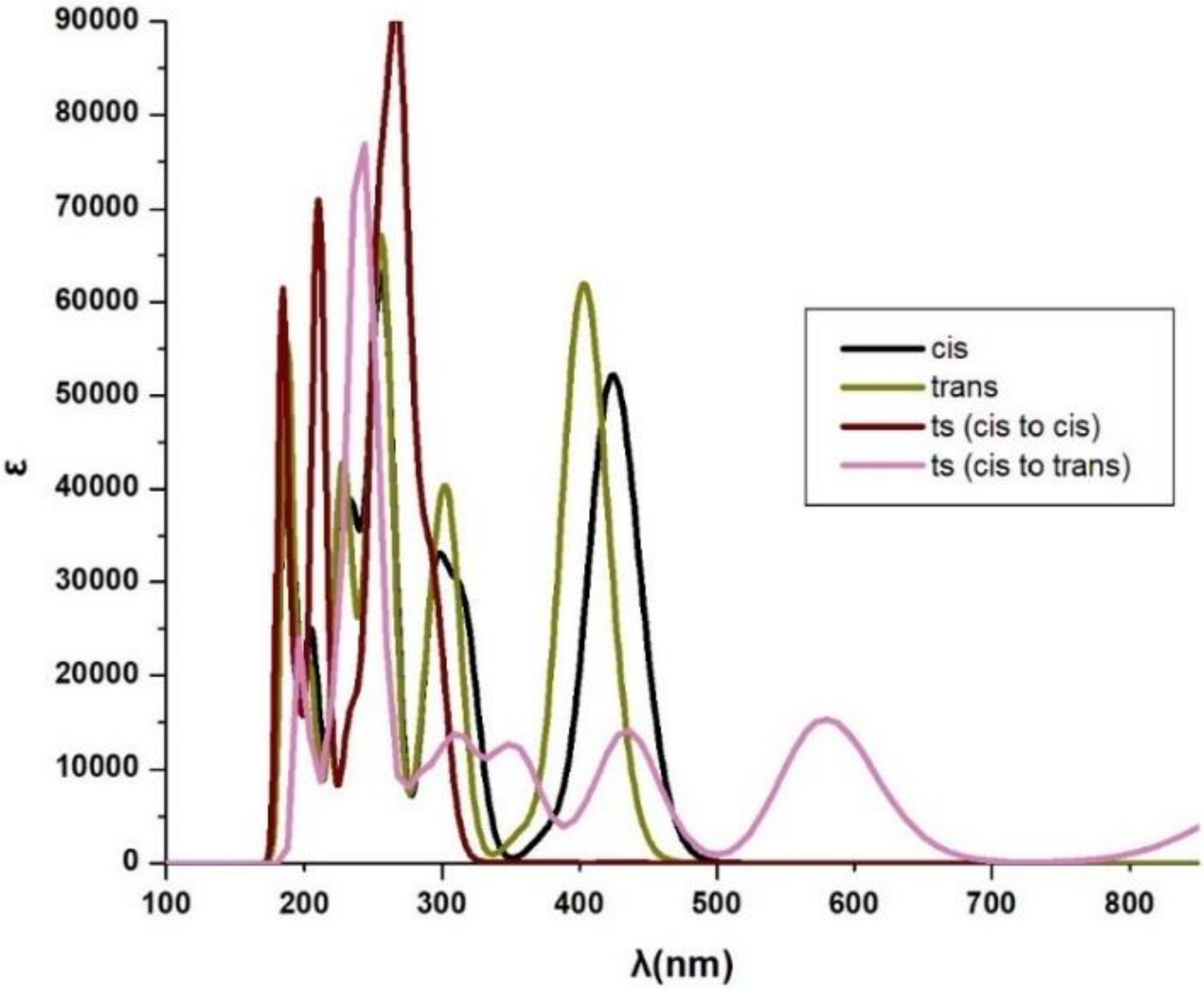

Figure S3: Absorption spectra of **1**-Ph at the PBE0/def2-TZVP level of theory in chloroform.

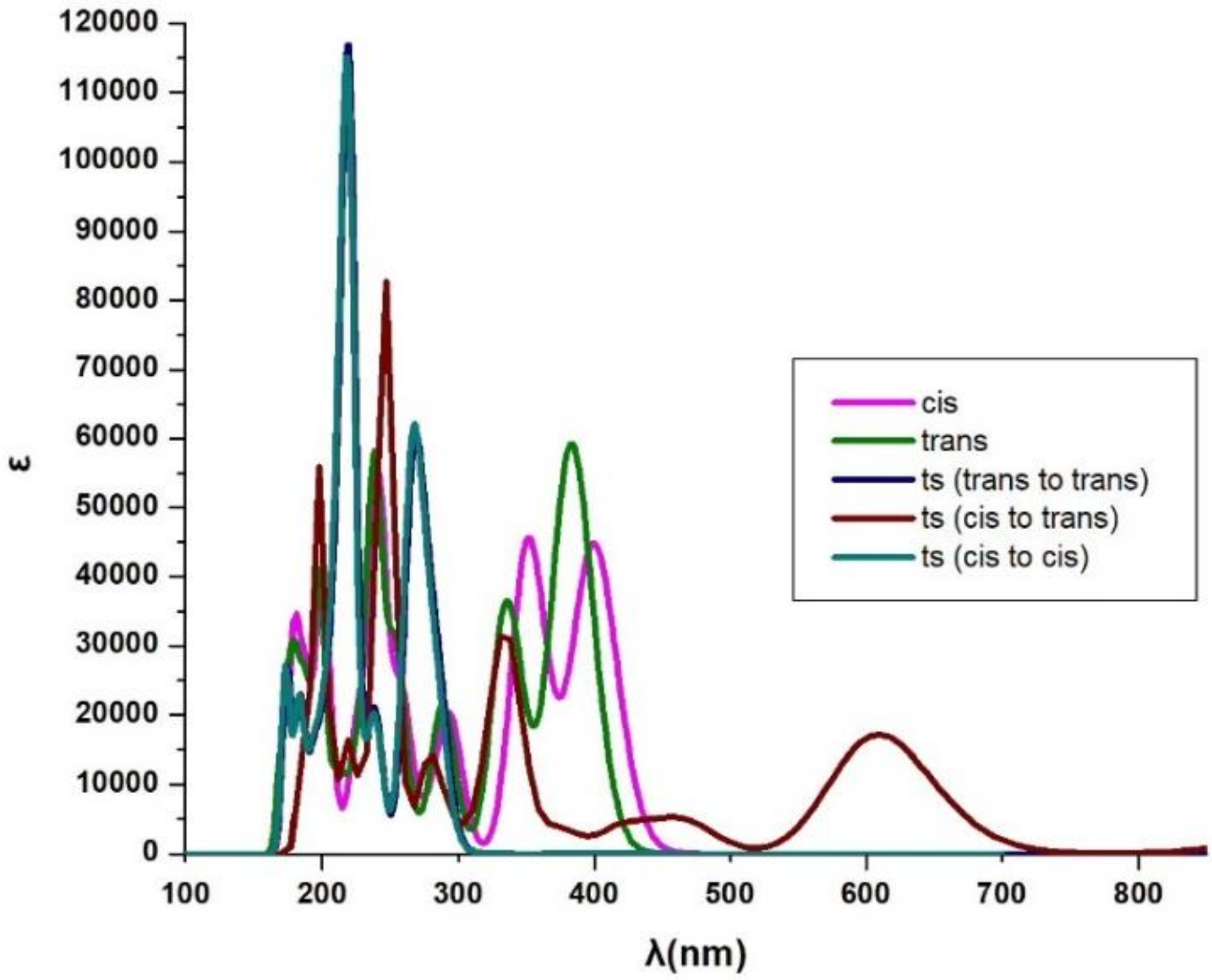

Figure S4: Absorption spectra of **1**-Na at the PBE0/def2-TZVP level of theory in chloroform.

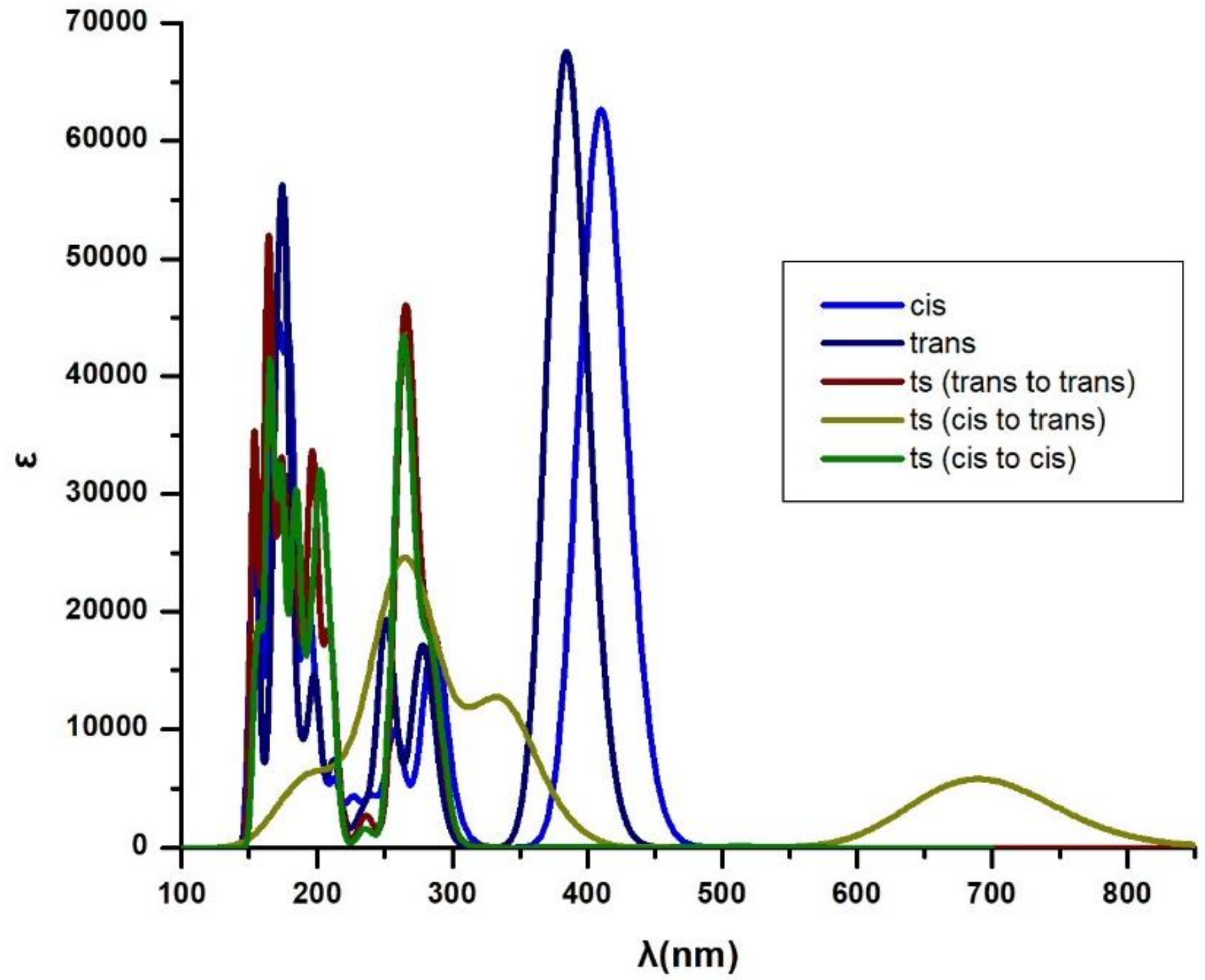

Figure S5: Absorption spectra of **1**-Pl at the PBE0/def2-TZVP level of theory in chloroform.

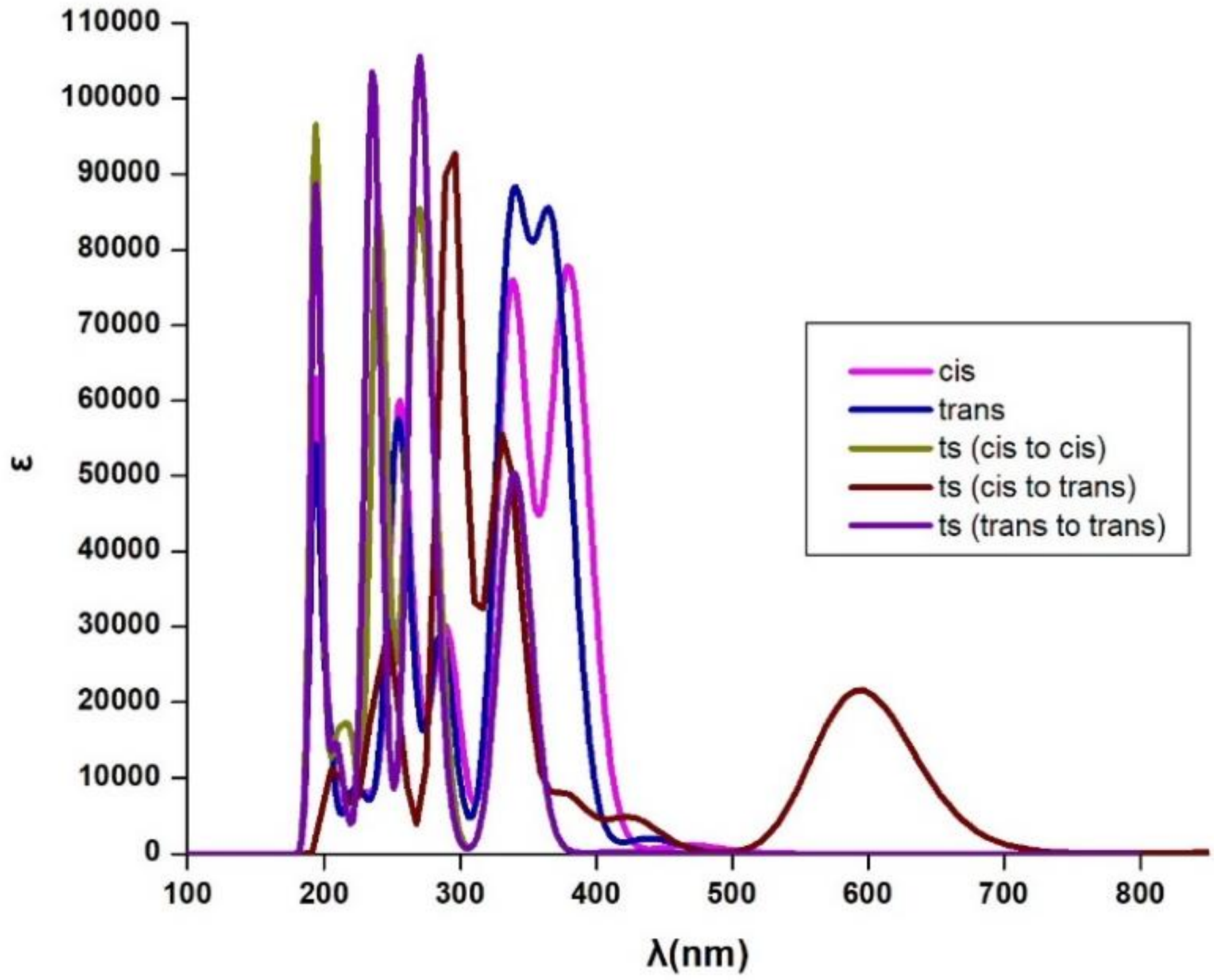

Figure S6: Absorption spectra of **1**-Pn at the PBE0/def2-TZVP level of theory in chloroform.

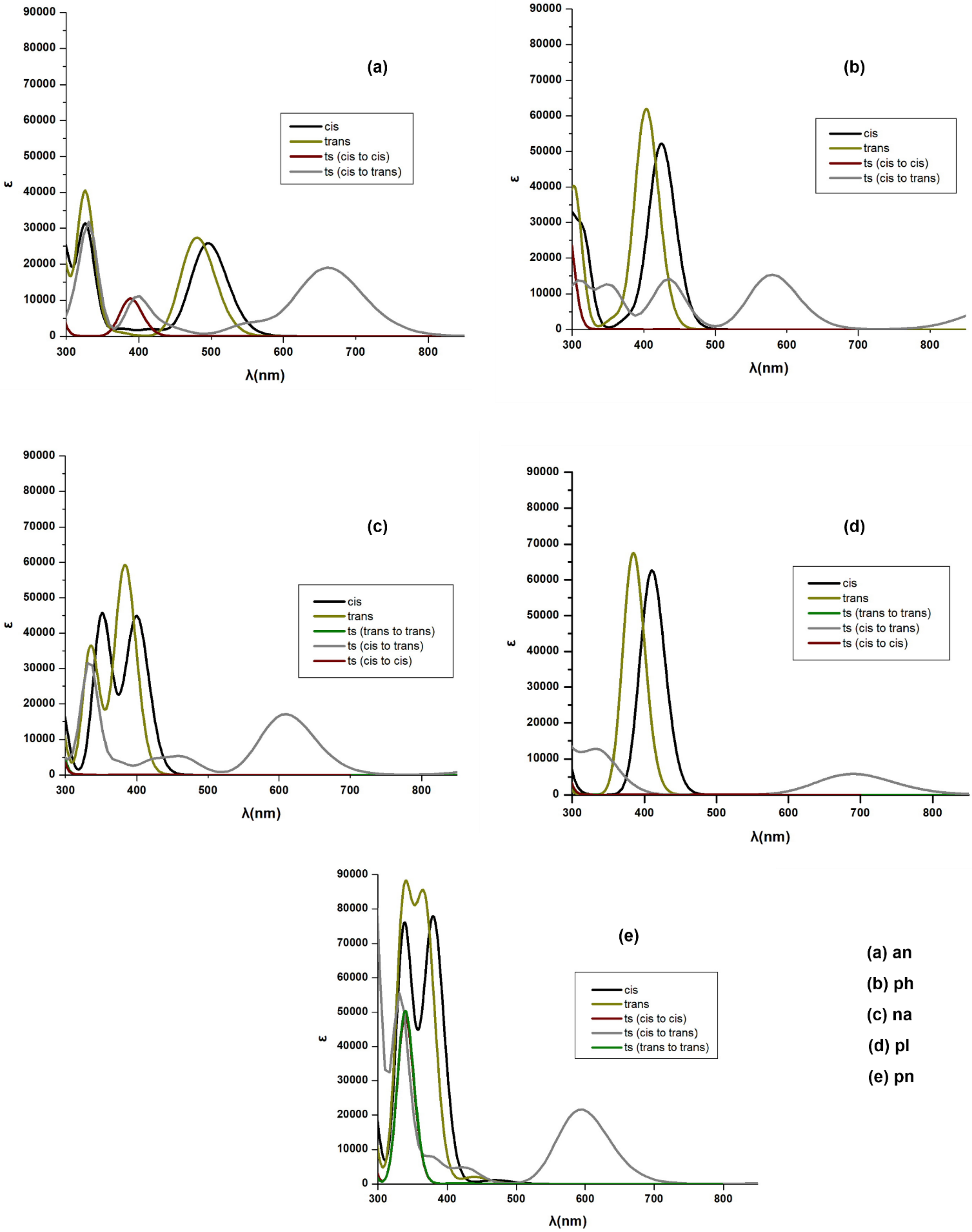

Figure S7: Near UV - vis absorption spectra of all molecules at the PBE0/def2-TZVP level of theory in chloroform.

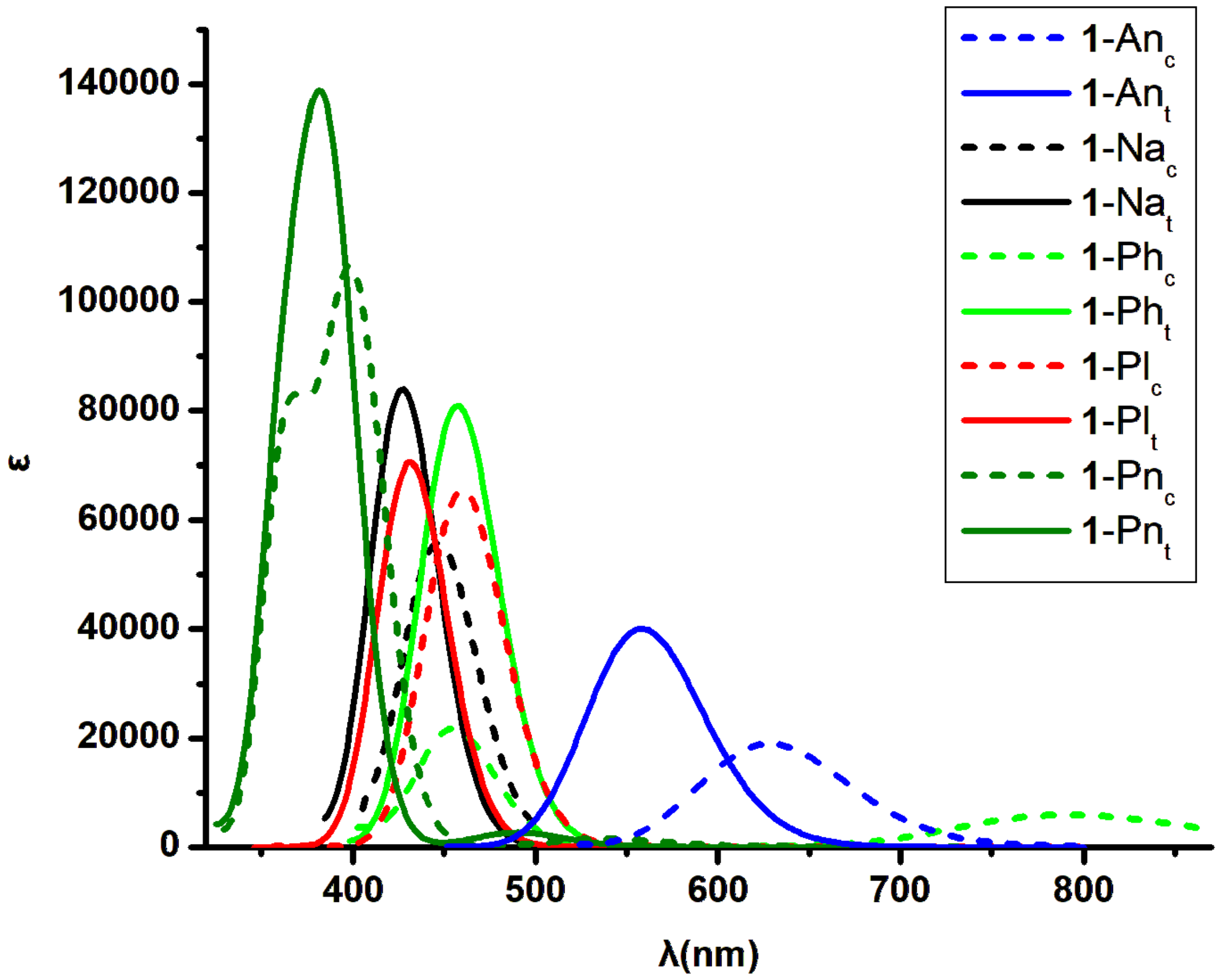

Figure S8: Emission spectra for all the molecules under study at PBE0/def2-TZVP in chloroform solvent.

Molecular Orbitals

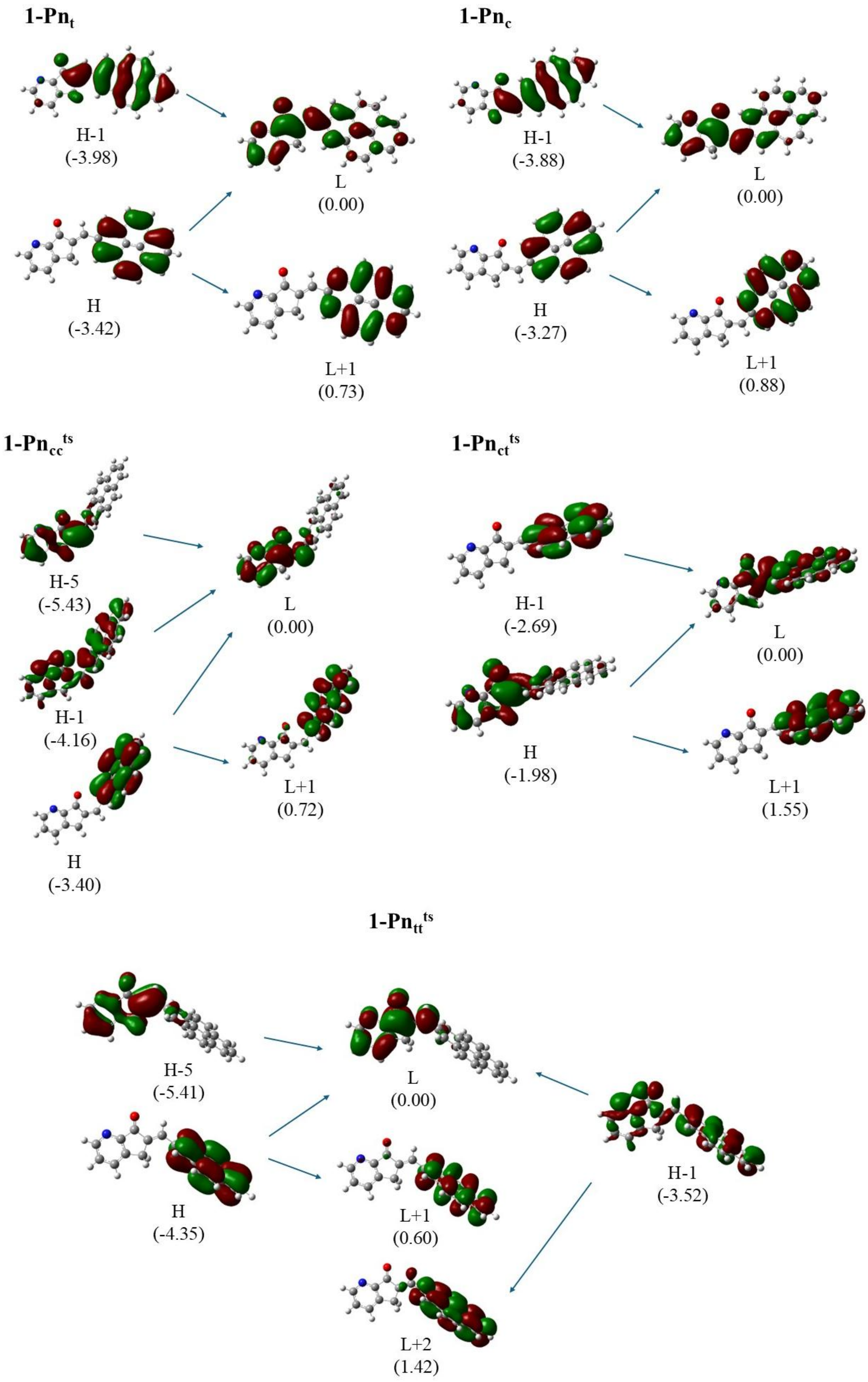

Figure S9: Electron density plots of the frontier molecular orbitals involved in the excitations corresponding to the main and the CT peaks of the **1-Pn** structures at the PBE0/def2-TZVP in chloroform solvent. Their relative energies of MO are given in eV.

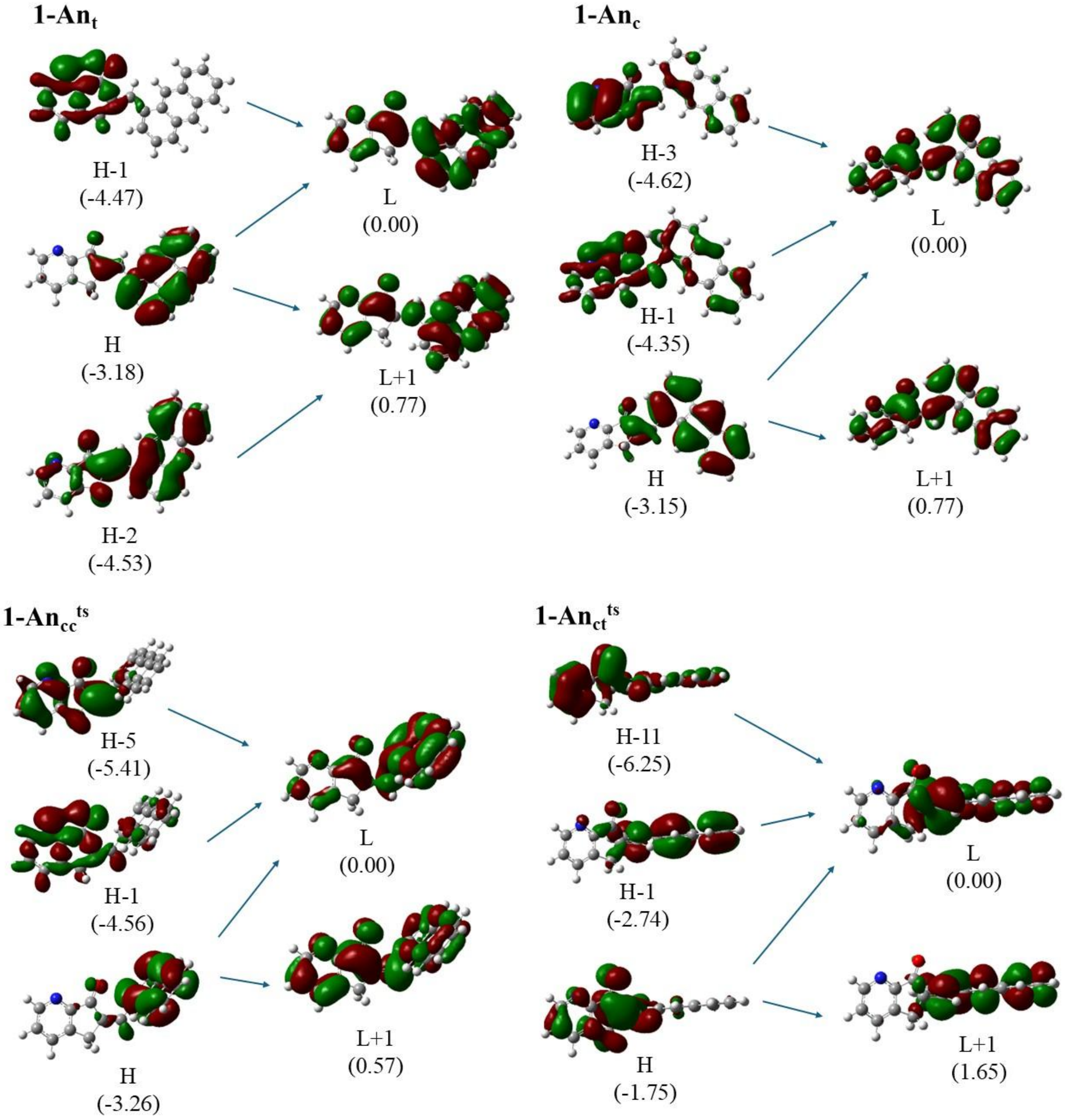

Figure S10: Electron density plots of the frontier molecular orbitals involved in the excitations corresponding to the main and the CT peaks of the **1-An** structures at the PBE0/def2-TZVP in chloroform solvent. Their relative energies of MO are given in eV.

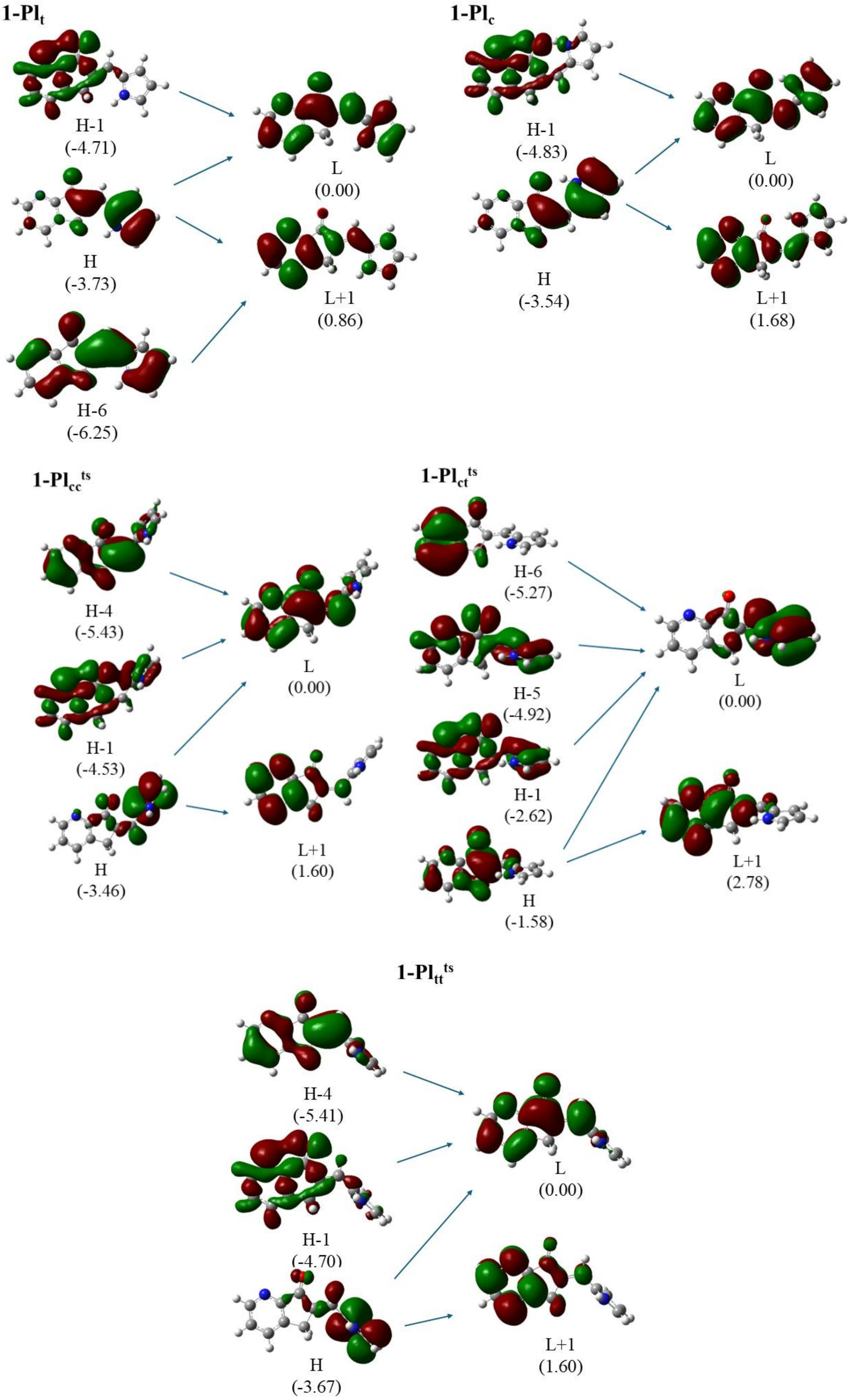

Figure S11: Electron density plots of the frontier molecular orbitals involved in the excitations corresponding to the main and the CT peaks of the **1-Pl** structures at the PBE0/def2-TZVP in chloroform solvent. Their relative energies of MO are given in eV.

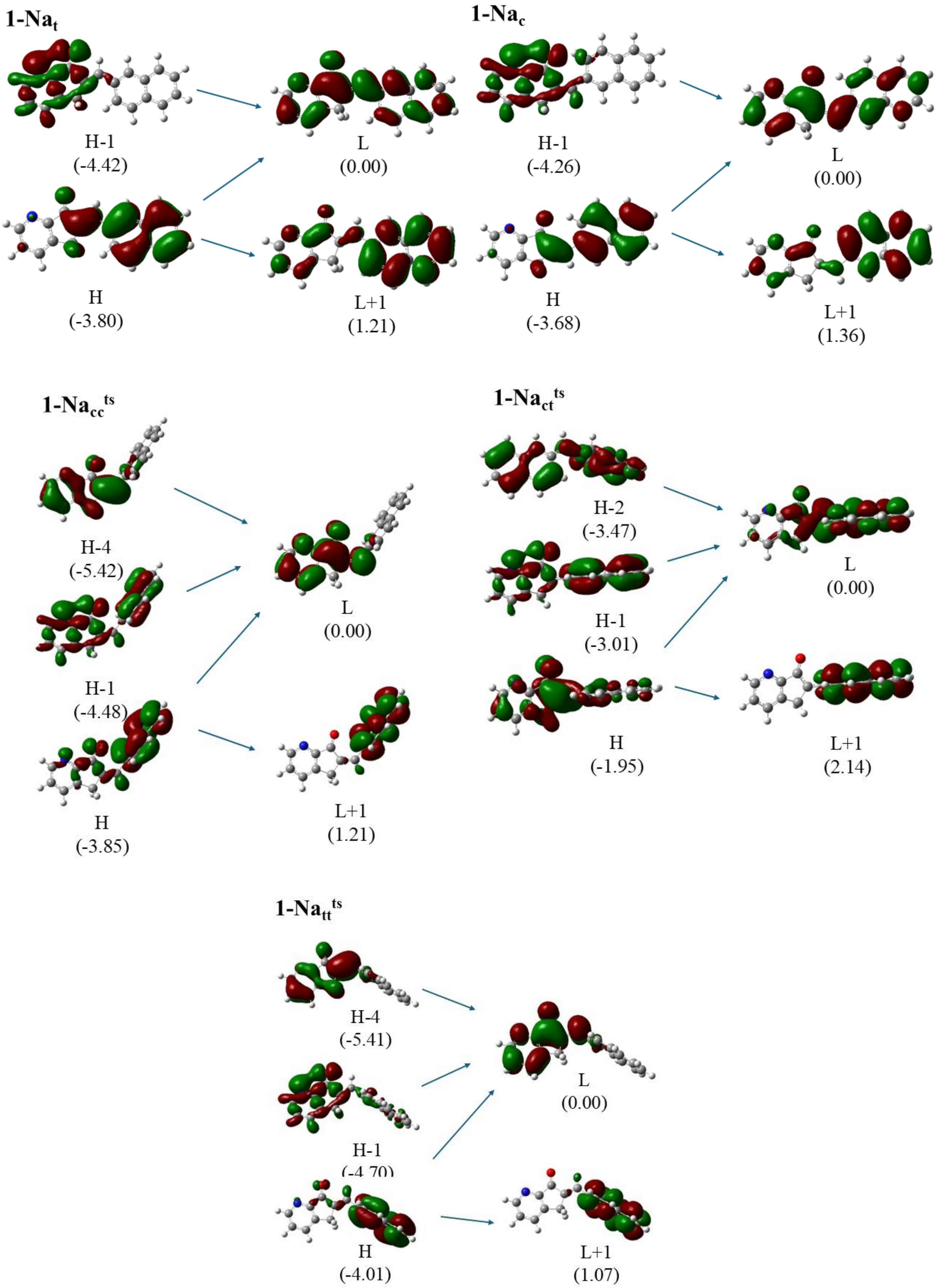

Figure S12: Electron density plots of the frontier molecular orbitals involved in the excitations corresponding to the main and the CT peaks of the **1-Na** structures at the PBE0/def2-TZVP in chloroform solvent. Their relative energies of MO are given in eV.

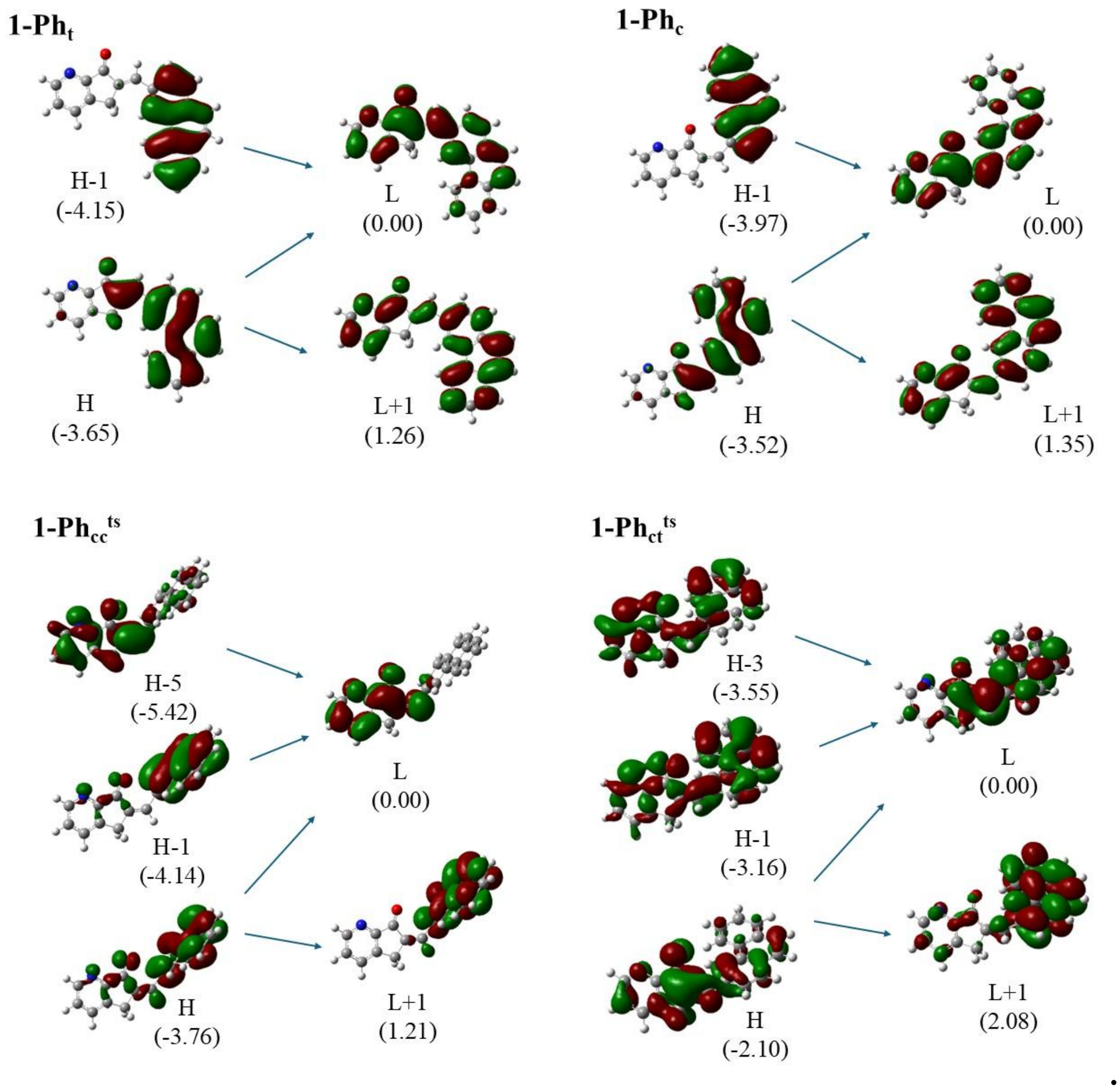

Figure S13: Electron density plots of the frontier molecular orbitals involved in the excitations corresponding to the main and the CT peaks of the **1-Ph** structures at the PBE0/def2-TZVP in chloroform solvent. Their relative energies of MO are given in eV.

Table S1. S_0 geometries of the cis-, trans- and transition state structures under study at the PBE0/def2-tzvp level of theory in chloroform solvent.

- **1**-An$_c$

0 1

C	-4.15384700	0.01805900	-0.34410200
C	-4.13160600	-1.13936900	0.43146100
C	-5.28310500	-1.91872600	0.48622600
C	-6.37940500	-1.48094900	-0.24654000
C	-6.28715600	-0.29780500	-0.99301800
N	-5.19512500	0.46014500	-1.05340400
H	-5.32835600	-2.83165700	1.07364200
H	-7.30763800	-2.04332200	-0.24846300
H	-7.14622200	0.04502900	-1.56643100
C	-2.84150700	0.72125400	-0.25682900
C	-1.97756500	-0.14210300	0.59594300
C	-2.79772900	-1.31992800	1.09163900
H	-2.34052700	-2.28026300	0.82388600
H	-2.89474200	-1.31144500	2.18488600
O	-2.57227200	1.78086000	-0.79877700
C	-0.65015300	-0.05068000	0.83778500
H	-0.23085800	-0.89408000	1.38912500
C	0.32714300	0.98049300	0.50404200
C	1.70164800	0.59265700	0.26487800
C	0.00261300	2.32107700	0.51978900
C	2.11251900	-0.73905600	0.17268600
C	2.69095700	1.62487700	0.08350600
C	0.97931500	3.32611400	0.32596500
H	-1.02495500	2.61071800	0.69780300
C	3.44900700	-1.09135700	-0.04901500
H	1.38413200	-1.54067300	0.25114100
C	4.02497800	1.27512900	-0.13968000
C	2.28865500	2.99074700	0.12263600
H	0.67742200	4.36851600	0.35464500
C	3.86633000	-2.45201100	-0.13341800
C	4.43525100	-0.05800300	-0.20372400
H	4.76061200	2.06504100	-0.27423500
H	3.04464500	3.75915300	-0.01615900
C	5.17861100	-2.76975400	-0.35057200
H	3.11963100	-3.23371700	-0.01964100
C	5.79346300	-0.42943700	-0.42959900
C	6.15488600	-1.74601600	-0.49961700
H	5.48400000	-3.81023400	-0.41096800
H	6.53520500	0.35649400	-0.54600200

H	7.19187400	-2.01872000	-0.67182800

- **1**-An_t

0 1

C	-4.16204200	-0.86986500	0.16104900
C	-4.17099200	0.40175000	-0.41237400
C	-5.33850600	0.83561000	-1.03169500
C	-6.41809400	-0.03965000	-1.03431400
C	-6.29517800	-1.29504400	-0.42359200
N	-5.18662600	-1.72559000	0.17446800
H	-5.40929400	1.81528100	-1.49620100
H	-7.35683300	0.23723500	-1.50331200
H	-7.14142900	-1.97914200	-0.42368300
C	-2.82186100	-1.13870200	0.75208000
C	-1.99714200	0.06574600	0.45589400
C	-2.85373100	1.10544600	-0.22356900
H	-2.99846200	1.99845600	0.39792500
H	-2.41854200	1.44984200	-1.16988700
O	-2.46276600	-2.14654300	1.33691900
C	-0.67396400	0.03805500	0.71115100
H	-0.33405300	-0.89773800	1.15445600
C	0.33622100	1.05693300	0.46106800
C	1.71059500	0.66502500	0.22597600
C	0.02173600	2.40100400	0.48943400
C	2.11739200	-0.66786100	0.13358000
C	2.70394700	1.69536600	0.05333300
C	1.00174500	3.40492900	0.30978500
H	-0.99690400	2.70482500	0.70141400
C	3.45294000	-1.02243100	-0.08783600
H	1.38974200	-1.46877400	0.21877500
C	4.03735200	1.34228900	-0.16968700
C	2.30876300	3.06297400	0.10210700
H	0.70649600	4.44871400	0.35257500
C	3.86522500	-2.38471500	-0.17307900
C	4.44256200	0.00824300	-0.23977000
H	4.77611900	2.13031900	-0.29748000
H	3.06896700	3.82844100	-0.02889600
C	5.17627300	-2.70610600	-0.39102200
H	3.11573600	-3.16353300	-0.05901500
C	5.79966500	-0.36729500	-0.46621300
C	6.15579600	-1.68503400	-0.53886900
H	5.47850200	-3.74738200	-0.45235500
H	6.54442200	0.41607300	-0.58022800
H	7.19184700	-1.96114500	-0.71132900

- **1**-An$_{cc}^{ts}$

0 1

C	3.40215800	-0.30306500	-0.58788500
C	3.93198600	-0.43968900	0.69485600
C	5.17631500	-1.04843800	0.83078700
C	5.80263600	-1.47789900	-0.33228600
C	5.17352500	-1.28719300	-1.57085700
N	3.98504300	-0.70804200	-1.71888300
H	5.64267600	-1.18456300	1.80264600
H	6.77421000	-1.96007400	-0.29494300
H	5.66467800	-1.62375200	-2.48169200
C	2.07191900	0.36593500	-0.51961500
C	1.83420600	0.64121100	0.93248000
C	3.00882400	0.12513000	1.73728700
H	3.49280900	0.92439700	2.31122800
H	2.70376200	-0.64231100	2.45897600
O	1.33882200	0.62464000	-1.45418900
C	0.75691800	1.25493200	1.44015800
H	0.71347400	1.38703400	2.52378900
C	-0.39152000	1.80035000	0.67684000
C	-1.51826500	0.96595700	0.35787300
C	-0.41510700	3.13115500	0.34573900
C	-1.56173300	-0.39170000	0.68400300
C	-2.64533600	1.55518500	-0.31810100
C	-1.52511000	3.70458900	-0.32967900
H	0.43486000	3.75894700	0.59752600
C	-2.66741300	-1.18876100	0.37398400
H	-0.71273000	-0.84549300	1.18811200
C	-3.74865000	0.75677700	-0.63070500
C	-2.60955700	2.94065100	-0.65238000
H	-1.50097800	4.75967000	-0.58554100
C	-2.71796300	-2.57493600	0.70669600
C	-3.79213600	-0.60000700	-0.30194900
H	-4.59594300	1.20612400	-1.14416300
H	-3.46313500	3.37427200	-1.16663900
C	-3.81197700	-3.33190100	0.39197800
H	-1.86593300	-3.01828300	1.21577800
C	-4.91416100	-1.42405800	-0.61337400
C	-4.92449500	-2.74888400	-0.27666400
H	-3.83782600	-4.38651700	0.65026600
H	-5.76183500	-0.97590000	-1.12544300
H	-5.78435600	-3.36642700	-0.51931500

- **1**-An$_{ct}^{ts}$

0 1

C	-4.24912800	-0.63601300	0.40955500
C	-4.25626500	-0.22039000	-0.92414600
C	-5.46223500	0.13384800	-1.50531500
C	-6.61008700	0.00520500	-0.72076900
C	-6.49043800	-0.45012500	0.59240400
N	-5.32907100	-0.76203500	1.17912800
H	-5.52215900	0.48589500	-2.53240200
H	-7.58724400	0.26549500	-1.11513000
H	-7.38165700	-0.55109700	1.21027800
C	-2.83526800	-0.79726500	0.90329400
C	-2.03091000	-0.43018200	-0.19853800
C	-2.85893000	-0.18845700	-1.45298900
H	-2.61987400	0.74499000	-1.98201400
H	-2.70809300	-1.00202200	-2.18380900
O	-2.52701900	-1.17021100	2.04963800
C	-0.60628600	-0.45024400	-0.34516400
H	-0.14814300	-1.33098900	-0.81188800
C	0.26633300	0.57627500	0.00871700
C	1.71777000	0.41652200	-0.01620900
C	-0.27809700	1.84917600	0.29407500
C	2.35932100	-0.80883900	-0.09743200
C	2.52342400	1.60759500	0.09878900
C	0.51852800	2.98501800	0.37648500
H	-1.35943600	1.92583700	0.38592200
C	3.76182300	-0.91018300	-0.12040400
H	1.78482800	-1.72976800	-0.09627400
C	3.91903900	1.51195200	0.08561500
C	1.89361000	2.86469900	0.26721500
H	0.06509100	3.96176300	0.50647000
C	4.41689500	-2.16819200	-0.20685900
C	4.56252200	0.27881900	-0.03287400
H	4.51155600	2.41816600	0.18350900
H	2.52135800	3.74982500	0.33130500
C	5.78496800	-2.24329500	-0.21434900
H	3.81311200	-3.06941300	-0.26576100
C	5.98261800	0.16157200	-0.04528200
C	6.57678400	-1.06707400	-0.13502200
H	6.27433000	-3.21055000	-0.27688200
H	6.58096200	1.06623400	0.01755000
H	7.65906800	-1.15098200	-0.14666000

- **1**-Na$_{c}$

0 1

C	-3.52850400	0.46934200	-0.00002500
C	-3.79770400	-0.89687300	0.00015200
C	-5.12726400	-1.30790100	0.00022400
C	-6.09657500	-0.31277200	0.00012300
C	-5.70792200	1.03456700	-0.00002700
N	-4.44184800	1.44349900	-0.00010900
H	-5.40088600	-2.35935800	0.00034600
H	-7.15229400	-0.56441700	0.00016900
H	-6.46783600	1.81354200	-0.00011100
C	-2.05700800	0.70639700	-0.00002800
C	-1.42639200	-0.64021900	0.00007300
C	-2.52817400	-1.69014700	0.00020500
H	-2.46532600	-2.34236300	-0.87989900
H	-2.46528600	-2.34222900	0.88040200
O	-1.53712700	1.81293300	-0.00009600
C	-0.12690000	-1.03012100	0.00007800
H	-0.00421900	-2.11519600	0.00018600
C	1.15896300	-0.34891000	0.00001800
C	2.28456900	-1.16517500	0.00000100
C	3.59378500	-0.63939400	-0.00004900
C	3.76540400	0.77768600	-0.00006900
C	2.60840300	1.59921000	-0.00003300
C	1.34724600	1.06541700	0.00000900
C	4.74126400	-1.47407100	-0.00007400
C	5.07727000	1.30877100	-0.00012600
C	6.17044500	0.47491400	-0.00015300
C	6.00151500	-0.92876100	-0.00012700
H	4.60530800	-2.55254500	-0.00005500
H	2.16315400	-2.24641500	0.00003300
H	0.47136900	1.70344800	-0.00003100
H	6.87475700	-1.57426800	-0.00015000
H	2.73765500	2.67869400	-0.00006000
H	5.20636900	2.38796800	-0.00014300
H	7.17266300	0.89341800	-0.00019400

- **1**-Na_t

0 1

C	3.75327700	0.50997600	0.00004900
C	3.38402800	-0.83451100	0.00013200
C	4.39103700	-1.79403300	0.00002500
C	5.70316100	-1.33599700	-0.00017200
C	5.95657300	0.04237000	-0.00025900
N	5.00518600	0.97335000	-0.00015200

H	4.16784900	-2.85744600	0.00007200
H	6.53525100	-2.03275600	-0.00027400
H	6.98395700	0.40111300	-0.00041700
C	2.53138600	1.36129200	0.00032000
C	1.37680500	0.42079000	0.00021200
C	1.88816000	-0.99926000	0.00027800
H	1.55471200	-1.56174900	0.88179900
H	1.55457900	-1.56185700	-0.88112400
O	2.48560900	2.58014600	-0.00006500
C	0.12500200	0.92305200	0.00014100
H	0.09135300	2.01376900	0.00014200
C	-1.17267900	0.27029700	0.00007700
C	-2.29994100	1.07989200	0.00004100
C	-3.60695900	0.54613100	-0.00002600
C	-3.77285000	-0.87177900	-0.00005800
C	-2.61326100	-1.68983900	-0.00002300
C	-1.35753800	-1.14449000	0.00004100
C	-4.75849600	1.37456800	-0.00006100
C	-5.08146500	-1.41021300	-0.00012500
C	-6.17869500	-0.58169700	-0.00015800
C	-6.01597700	0.82239500	-0.00012500
H	-4.62807100	2.45354000	-0.00003600
H	-2.17902000	2.16083800	0.00006600
H	-0.49814600	-1.80286300	0.00006000
H	-6.89224900	1.46369400	-0.00015200
H	-2.73685000	-2.76970000	-0.00005100
H	-5.20507800	-2.49001600	-0.00015000
H	-7.17886000	-1.00484500	-0.00020900

- **1**-Na_{cc}^{ts}

0 1

C	-3.11633200	-0.56570200	-0.21184500
C	-3.75199300	0.53276200	0.36598400
C	-5.13450700	0.48583900	0.52088100
C	-5.78301400	-0.66245900	0.08465200
C	-5.03913200	-1.70717400	-0.48221900
N	-3.71827900	-1.67885700	-0.63831700
H	-5.68809000	1.30944700	0.96326000
H	-6.85981200	-0.76185300	0.17670900
H	-5.54760400	-2.60665400	-0.82362800
C	-1.64798700	-0.31949300	-0.29131100
C	-1.43404900	1.03500900	0.30825600
C	-2.77076400	1.61174900	0.72695700
H	-2.79982200	1.83786500	1.79955400

H	-2.99414700	2.54735700	0.20005600
O	-0.81097400	-1.06727800	-0.75886400
C	-0.25105700	1.64711700	0.45020500
H	-0.24873700	2.64740000	0.88936000
C	1.07614800	1.10955900	0.06591300
C	1.82480200	0.37507800	0.95874500
C	3.12018700	-0.08939300	0.62346800
C	3.65770200	0.22000500	-0.66185500
C	2.87178300	0.98349600	-1.56058200
C	1.61953500	1.41895200	-1.20862100
C	3.90178700	-0.85249600	1.52761500
C	4.95500800	-0.24359600	-0.99475300
C	5.68830100	-0.98102300	-0.09725500
C	5.15606800	-1.28821700	1.17593800
H	3.48956200	-1.08906200	2.50529800
H	1.42055100	0.13662900	1.93930200
H	1.02709500	2.00120500	-1.90830400
H	5.74478500	-1.87319300	1.87661600
H	3.27632200	1.21960000	-2.54155500
H	5.35969700	-0.00623800	-1.97544400
H	6.68102700	-1.33235500	-0.36312400

- **1**-Na_{ct}^{ts}

0 1

C	-3.44750000	-0.22944100	0.44798200
C	-3.53897800	0.55637400	-0.70299700
C	-4.71803400	0.54909800	-1.42438100
C	-5.75497200	-0.25992200	-0.94812000
C	-5.56040700	-0.99348200	0.21870200
N	-4.42344800	-0.99346800	0.92911800
H	-4.84163800	1.14574100	-2.32507400
H	-6.70424000	-0.31683300	-1.47137600
H	-6.36471700	-1.61898300	0.60349700
C	-2.09531000	-0.06243300	1.10523100
C	-1.37927900	0.75826700	0.18792900
C	-2.26996500	1.32560800	-0.88759700
H	-2.46939200	2.39684700	-0.70300300
H	-1.85978100	1.26011100	-1.90521600
O	-1.74198500	-0.66868000	2.13486500
C	-0.05287200	1.27064200	0.42783000
H	0.07614600	2.30011600	0.79591500
C	1.11477800	0.57925600	0.15291200
C	2.36279100	1.19435800	0.40897000
C	3.56030400	0.53293100	0.13897600

C	3.50799800	-0.79888000	-0.39934500
C	2.24102000	-1.41216800	-0.64661900
C	1.08095000	-0.76123800	-0.36600300
C	4.82222700	1.14562900	0.38055800
C	4.71990200	-1.46554000	-0.65786000
C	5.92710800	-0.84424000	-0.41258600
C	5.98243100	0.46895800	0.10744700
H	4.84512100	2.15463900	0.78207900
H	2.38199500	2.20351700	0.81378400
H	0.10574800	-1.21269600	-0.51765800
H	6.94537900	0.93499900	0.28810500
H	2.22619100	-2.42227100	-1.04563600
H	4.69299100	-2.47383300	-1.05965100
H	6.85306600	-1.37029700	-0.62470700

- **1**-Na_{tt}^{ts}

0 1

C	-3.64907200	0.25534900	-0.16117700
C	-2.97350800	-0.91962400	0.17326300
C	-3.72377300	-2.07779200	0.35502900
C	-5.09917300	-1.97645000	0.18854300
C	-5.66921700	-0.74061000	-0.14872100
N	-4.96869700	0.37638900	-0.32644400
H	-3.25829000	-3.02412800	0.61622900
H	-5.73972500	-2.84317900	0.31636800
H	-6.74667800	-0.66207500	-0.27897400
C	-2.67142200	1.36874600	-0.30913900
C	-1.33213300	0.76693800	-0.03528700
C	-1.48867400	-0.70106700	0.27903500
H	-0.93453100	-1.33133200	-0.42686700
H	-1.11307100	-0.94922500	1.27910200
O	-2.90581500	2.52881600	-0.59326200
C	-0.21136600	1.49465300	-0.08839200
H	-0.32307400	2.55113000	-0.33937800
C	1.14703200	0.97196400	0.17691300
C	1.93772800	0.50097600	-0.84923500
C	3.24125600	0.00320800	-0.60327300
C	3.73954700	-0.00665200	0.73419100
C	2.90939200	0.48400500	1.77269900
C	1.65049700	0.96018300	1.50528200
C	4.06706400	-0.48656200	-1.64677200
C	5.04396600	-0.50456200	0.97858000
C	5.82043000	-0.97233000	-0.05340400
C	5.32702300	-0.96297100	-1.37827500

H	3.68580100	-0.47736000	-2.66470400
H	1.56334100	0.50838200	-1.86995200
H	1.02496000	1.33554900	2.30997000
H	5.95109000	-1.33483100	-2.18563300
H	3.28544300	0.47811900	2.79260500
H	5.41984200	-0.50936300	1.99856500
H	6.81871600	-1.35134500	0.14471200

- **1**-Ph$_c$

0 1

C	-3.76800400	0.69687000	0.00042600
C	-4.54119800	-0.46147600	0.00045800
C	-5.92664000	-0.33045900	0.00057900
C	-6.43944500	0.96069800	0.00065500
C	-5.56327000	2.05554600	0.00060900
N	-4.23718700	1.94712100	0.00049700
H	-6.58324500	-1.19615400	0.00061200
H	-7.51086300	1.13363100	0.00074900
H	-5.96580200	3.06661500	0.00066700
C	-2.31918600	0.35004100	0.00029800
C	-2.25336700	-1.13333600	0.00023600
C	-3.67297700	-1.68098100	0.00034900
H	-3.86501300	-2.30735200	-0.87983200
H	-3.86485600	-2.30740100	0.88052900
O	-1.41335500	1.17360900	0.00026300
C	-1.19963400	-1.98997300	0.00009600
C	4.51197400	3.11195100	-0.00035800
C	5.17895500	1.90615300	-0.00051700
C	4.46977100	0.68641200	-0.00048300
C	3.04836900	0.69895100	-0.00028500
C	2.39210700	1.94761600	-0.00012400
C	3.10773400	3.12827700	-0.00015900
C	5.16904700	-0.56319200	-0.00064200
C	2.33314400	-0.56748700	-0.00025700
C	3.07289100	-1.78227200	-0.00041200
C	4.50138900	-1.74651800	-0.00060500
C	2.37689500	-3.01117500	-0.00037700
H	2.94768600	-3.93599100	-0.00049200
C	1.00519000	-3.04554400	-0.00020400
C	0.24714300	-1.84492200	-0.00005900
C	0.93012100	-0.62941400	-0.00008500
H	6.25589900	-0.54485900	-0.00079200
H	5.06731100	4.04514300	-0.00038600
H	6.26566600	1.87767800	-0.00066900

H	1.30800700	1.99273500	0.00003200
H	2.57771200	4.07617300	-0.00003200
H	5.04413700	-2.68799800	-0.00072400
H	0.48742700	-4.00128600	-0.00018200
H	0.33678500	0.27573400	0.00002800
H	-1.49975500	-3.03999400	0.00008600

- **1**-Ph_t

0 1

C	4.37663300	-0.06738200	-0.00000500
C	3.51183100	1.02638400	0.00009900
C	4.06429700	2.30272300	0.00022100
C	5.45121200	2.39319200	0.00020000
C	6.22257400	1.22316900	0.00005100
N	5.71009600	-0.00543300	-0.00004700
H	3.44362200	3.19452600	0.00033500
H	5.94531800	3.35945700	0.00031300
H	7.30843500	1.29398300	0.00005500
C	3.58370200	-1.32811700	-0.00007000
C	2.15380800	-0.91227200	-0.00003700
C	2.07013100	0.59333000	0.00006800
H	1.54218400	0.97978800	-0.88181500
H	1.54212900	0.97971200	0.88194300
O	4.01590000	-2.46840700	-0.00015500
C	1.19539500	-1.86125100	-0.00009400
C	-4.64928600	3.09539700	-0.00006400
C	-5.28090500	1.87089000	0.00016100
C	-4.53878000	0.67101900	0.00014100
C	-3.11783000	0.72099200	-0.00007500
C	-2.50068200	1.98898900	-0.00034900
C	-3.24650200	3.15061500	-0.00034100
C	-5.20738900	-0.59508800	0.00028200
C	-2.36912700	-0.52689100	-0.00003900
C	-3.08025800	-1.75951300	0.00003000
C	-4.50943700	-1.75972900	0.00018500
C	-2.35930000	-2.97288700	-0.00000300
H	-2.91157900	-3.90870900	0.00003200
C	-0.98696500	-2.98046000	-0.00006900
C	-0.25493200	-1.76703600	-0.00006900
C	-0.96440100	-0.56657100	-0.00004200
H	-6.29412200	-0.60361000	0.00044800
H	-5.23072500	4.01238100	-0.00004400
H	-6.36627500	1.81143000	0.00033900
H	-1.41888900	2.06891900	-0.00061900

H	-2.74262100	4.11259400	-0.00056900
H	-5.02657500	-2.71536500	0.00024200
H	-0.44598700	-3.92266300	-0.00008800
H	-0.41524600	0.36456600	0.00001800
H	1.58972400	-2.87876000	-0.00011800

- **1**-Ph_{cc}^{ts}

0 1

C	3.81964400	0.13377900	0.46761500
C	3.69254900	0.19189400	-0.92211900
C	4.53230200	1.03679300	-1.63140200
C	5.45780100	1.77790600	-0.89727200
C	5.49832100	1.64018300	0.49197900
N	4.69571700	0.83100900	1.18927700
H	4.47759200	1.12301100	-2.71362100
H	6.14458000	2.45763800	-1.39145900
H	6.21933800	2.21583900	1.07000400
C	2.82000600	-0.82857200	1.04301000
C	2.07237900	-1.30759700	-0.09390400
C	2.60580400	-0.73039200	-1.38847900
H	3.00503900	-1.49929200	-2.06664600
H	1.83013400	-0.19677000	-1.95605300
O	2.71413000	-1.11776600	2.23965200
C	1.06626000	-2.31114200	-0.03739500
C	-3.90093200	3.42434200	0.18115800
C	-4.71198000	2.31963800	0.03829800
C	-4.16016400	1.02303400	-0.02837300
C	-2.75140200	0.84990000	0.04714200
C	-1.94806600	1.99662200	0.19439200
C	-2.50966700	3.25695100	0.26126600
C	-5.00794800	-0.11927600	-0.16724700
C	-2.20526200	-0.49858300	-0.02558700
C	-3.09881700	-1.60883500	-0.14977100
C	-4.50068100	-1.38258100	-0.22271000
C	-2.58194600	-2.92999200	-0.19552300
H	-3.28291100	-3.75566900	-0.27904400
C	-1.23849500	-3.16611900	-0.13060600
C	-0.32442700	-2.07302000	-0.04174900
C	-0.83675400	-0.76045600	0.01520400
H	-6.08145500	0.04069800	-0.22372000
H	-4.33329300	4.41867900	0.23470700
H	-5.79114200	2.43138800	-0.02313900
H	-0.86995300	1.90445000	0.26521300
H	-1.86660100	4.12380400	0.37913600

H	-5.16207500	-2.23813000	-0.32384700
H	-0.84822100	-4.17933800	-0.16251300
H	-0.11073800	0.04053700	0.10775300
H	1.35550600	-3.36862700	-0.00649100

- **1**-Ph_{ct}^{ts}

0 1

C	-3.55630600	0.37073500	0.44680900
C	-3.89559700	-0.20986400	-0.77438700
C	-4.99581900	0.27110200	-1.46289100
C	-5.70964900	1.31737800	-0.87221100
C	-5.28663100	1.81816200	0.35790200
N	-4.21942100	1.36759100	1.03068800
H	-5.29967800	-0.14494600	-2.42087000
H	-6.58380900	1.74101900	-1.35674900
H	-5.83924200	2.63191400	0.82605400
C	-2.38096400	-0.34864400	1.04408300
C	-1.94562100	-1.26358400	0.05215500
C	-2.94379700	-1.30805200	-1.11088200
H	-2.52529600	-1.13819300	-2.11395600
H	-3.47565900	-2.27118800	-1.15991100
O	-1.85439900	-0.04817200	2.13878400
C	-1.03337500	-2.32913700	0.25117600
C	3.73126800	3.44446400	-0.17710900
C	4.57189000	2.35553700	-0.24540600
C	4.05146800	1.04524800	-0.18699600
C	2.65108900	0.84266700	-0.05384800
C	1.81587800	1.97183600	0.00660300
C	2.34683600	3.24633900	-0.05173800
C	4.91811800	-0.08353400	-0.26316900
C	2.14828900	-0.52142500	-0.00155300
C	3.06233400	-1.62297000	-0.09199200
C	4.44870600	-1.36755500	-0.21641900
C	2.58760100	-2.97025300	-0.03210900
H	3.31501300	-3.77312100	-0.10171000
C	1.26234100	-3.24097200	0.10065000
C	0.33068300	-2.15395400	0.16937100
C	0.80354000	-0.81735500	0.17191800
H	5.98541500	0.09622300	-0.36352000
H	4.13365700	4.45132800	-0.22287300
H	5.64515300	2.48986800	-0.34601200
H	0.74133400	1.85659400	0.09278600
H	1.68226100	4.10325200	-0.00228100
H	5.13543700	-2.20605700	-0.27572800

H	0.89203500	-4.26178500	0.12882700
H	0.03582600	-0.06256200	0.31721700
H	-1.36499600	-3.37711400	0.31389500

- **1**-Pn_c

0 1

C	4.70156600	-0.57694300	-0.00010700
C	5.17066500	0.73423300	-0.00005200
C	6.54659200	0.94322700	-0.00005900
C	7.35698500	-0.18506000	-0.00012400
C	6.77242400	-1.45985500	-0.00017600
N	5.45972600	-1.67631200	-0.00016900
H	6.97333500	1.94239000	-0.00001700
H	8.43839100	-0.09329500	-0.00013300
H	7.40833300	-2.34297800	-0.00022700
C	3.21179200	-0.59183300	-0.00009200
C	2.78806500	0.83333200	-0.00000900
C	4.03306400	1.70771200	0.00001200
H	4.06795400	2.36210400	-0.87999900
H	4.06798200	2.36202300	0.88008100
O	2.53137900	-1.60769000	-0.00010900
C	1.55972200	1.40778300	0.00003900
H	1.59541500	2.49916300	0.00008800
C	0.18715000	0.91781500	0.00003800
C	-0.81801100	1.90241300	0.00008300
C	-2.17371300	1.57532600	0.00008900
C	-2.54991900	0.20297600	0.00005100
C	-1.54135200	-0.80117400	0.00000600
C	-0.19409000	-0.43402000	0.00000000
C	-3.20562800	2.57343300	0.00013400
C	-3.92335500	-0.16053000	0.00005700
C	-4.92671200	0.84980100	0.00010100
C	-4.51858000	2.22587200	0.00014000
C	-6.27389500	0.46781600	0.00010600
H	-7.04295400	1.23564800	0.00014000
C	-6.62922800	-0.87721500	0.00006900
C	-5.65429100	-1.86968300	0.00002500
C	-4.29452800	-1.53517300	0.00001800
C	-3.25828000	-2.52840500	-0.00002600
C	-1.94578700	-2.17947500	-0.00003200
H	-1.17148200	-2.94169500	-0.00006600
H	-3.54864000	-3.57574800	-0.00005500
H	-2.91266800	3.62002900	0.00016300
H	-0.53429400	2.95252100	0.00011400

H	0.57648800	-1.19739600	-0.00003700
H	-5.28966200	2.99180100	0.00017400
H	-7.67903100	-1.15552100	0.00007400
H	-5.94223000	-2.91757800	-0.00000400

- **1**-Pn_t

0 1

C	-5.07889800	0.33967600	-0.00005400
C	-4.50861200	-0.93264200	-0.00000500
C	-5.35734800	-2.03461700	-0.00000300
C	-6.72396900	-1.78225300	-0.00005600
C	-7.18499900	-0.45872900	-0.00008700
N	-6.38694800	0.60655200	-0.00009700
H	-4.97434100	-3.05139500	0.00003200
H	-7.43987700	-2.59792700	-0.00007000
H	-8.25508400	-0.26120600	-0.00014900
C	-4.00116000	1.36747000	-0.00002100
C	-2.71601700	0.61319700	0.00003600
C	-3.00489900	-0.86730600	0.00003100
H	-2.58856600	-1.37180200	-0.88154500
H	-2.58859400	-1.37181500	0.88160900
O	-4.14029000	2.57882400	-0.00004200
C	-1.55556300	1.29885200	0.00010100
H	-1.68737200	2.38196000	0.00009900
C	-0.17152100	0.84955100	0.00011000
C	0.82439300	1.83933900	0.00009100
C	2.18288900	1.52001300	0.00005700
C	2.56912800	0.15055200	0.00004400
C	1.56910800	-0.86322900	0.00009100
C	0.22136400	-0.49882400	0.00012400
C	3.20671100	2.52593000	0.00001600
C	3.94537000	-0.20247200	-0.00001000
C	4.94053800	0.81566700	-0.00006000
C	4.52230300	2.18820100	-0.00004300
C	6.29093800	0.44435700	-0.00012000
H	7.05341700	1.21866400	-0.00016300
C	6.65768700	-0.89729400	-0.00012500
C	5.69052600	-1.89754900	-0.00007000
C	4.32855900	-1.57354300	-0.00001300
C	3.30067900	-2.57516900	0.00004400
C	1.98536300	-2.23774900	0.00009400
H	1.21999800	-3.00925000	0.00013600
H	3.59954700	-3.62008200	0.00004600
H	2.90519600	3.56989800	0.00003400

H	0.52864900	2.88565900	0.00011000
H	-0.52120500	-1.28778700	0.00017700
H	5.28734500	2.96005200	-0.00008000
H	7.70965400	-1.16691600	-0.00017100
H	5.98677800	-2.94308300	-0.00007200

- **1**-Pn_{cc}^{ts}

0 1

C	-4.34624000	-0.00065100	0.72013700
C	-5.08271600	0.00065400	-0.46397900
C	-6.47179200	-0.00033200	-0.37439700
C	-7.02514700	-0.00251500	0.89966000
C	-6.18437300	-0.00359300	2.02187300
N	-4.85575200	-0.00270600	1.95447500
H	-7.10116100	0.00056500	-1.26010400
H	-8.10154400	-0.00339400	1.03827200
H	-6.61819900	-0.00526700	3.01984700
C	-2.88762600	0.00052200	0.41171200
C	-2.79237900	0.00241000	-1.08222600
C	-4.18802500	0.00313700	-1.67093900
H	-4.36537600	0.88468300	-2.29869400
H	-4.36492800	-0.87590400	-2.30229700
O	-1.97524500	0.00000100	1.21527000
C	-1.65578900	0.00290700	-1.79062600
H	-1.73639500	0.00407500	-2.87986500
C	-0.27545900	0.00199100	-1.24737700
C	0.39064800	1.20817800	-1.02539800
C	1.71417200	1.23064800	-0.57220900
C	2.39049900	0.00041500	-0.34377900
C	1.71363500	-1.22901300	-0.57492600
C	0.39012400	-1.20496800	-1.02808400
C	2.41831500	2.45743800	-0.33154500
C	3.73743200	-0.00038800	0.11303000
C	4.41194800	1.23180800	0.34310000
C	3.70481300	2.45729000	0.10518300
C	5.73829400	1.20676500	0.79270400
H	6.25519500	2.14631300	0.96967100
C	6.39063500	-0.00196300	1.01248200
C	5.73776900	-1.20991500	0.79002000
C	4.41140900	-1.23338500	0.34036600
C	3.70373400	-2.45802700	0.09975400
C	2.41723300	-2.45664000	-0.33696400
H	1.89498100	-3.39339200	-0.51291000
H	4.22283400	-3.39654700	0.27632200
H	1.89648700	3.39480900	-0.50544300

H	-0.12656300	2.14797300	-1.19962100
H	-0.12747100	-2.14415600	-1.20441300
H	4.22433200	3.39518700	0.28381400
H	7.41907300	-0.00257400	1.36160900
H	6.25426300	-2.15007800	0.96489900

- **1**-Pn_{ct}^{ts}

0 1

C	-4.63663500	-0.16214100	0.51365800
C	-4.77083100	0.14295800	-0.84355200
C	-5.90608300	-0.27932000	-1.51151000
C	-6.89228900	-0.91971100	-0.75456400
C	-6.67223300	-1.13495700	0.60336900
N	-5.55105400	-0.78774500	1.25039200
H	-6.02724000	-0.13423400	-2.58239100
H	-7.82307300	-1.24331000	-1.20959100
H	-7.43643900	-1.62882300	1.20198400
C	-3.30165500	0.30693700	1.03478400
C	-2.69271900	0.93660900	-0.08143200
C	-3.52872000	0.80544100	-1.34331900
H	-3.73214700	1.77329300	-1.82620100
H	-3.06109200	0.17167400	-2.11379300
O	-2.87482900	0.08612000	2.18379900
C	-1.48622900	1.70749100	-0.07969400
H	-1.54900500	2.80727100	-0.06774600
C	-0.18258600	1.20583700	-0.04118400
C	0.91858100	2.10325500	0.02475200
C	2.21984000	1.63203400	-0.00134000
C	2.44535700	0.21656600	-0.05789200
C	1.34035400	-0.69641200	-0.10235600
C	0.05076000	-0.19433800	-0.09946000
C	3.35204300	2.51619300	0.06294400
C	3.76926400	-0.28344800	-0.05692800
C	4.87263000	0.61760300	0.02531100
C	4.61671300	2.02938400	0.08021700
C	6.17070300	0.09920400	0.03450000
H	7.01381000	0.78012900	0.10688600
C	6.38553800	-1.27341500	-0.04792600
C	5.31667100	-2.16002600	-0.13305200
C	3.99981900	-1.68990300	-0.13005600
C	2.87440000	-2.58007100	-0.17200200
C	1.60378000	-2.10894600	-0.14734600
H	0.75752600	-2.78918300	-0.15259500
H	3.06471800	-3.64876200	-0.21045400

H	3.16885100	3.58626400	0.08726500
H	0.72653800	3.16664600	0.14229900
H	-0.81029800	-0.85475700	-0.14498700
H	5.46584000	2.70473700	0.13280100
H	7.40090500	-1.65788800	-0.04164900
H	5.49830000	-3.22874600	-0.20050900

- **1**-Pn_{tt}^{ts}

0 1

C	-4.96787000	0.00004600	-0.11653500
C	-4.18738800	-0.00039800	1.04091200
C	-4.83917900	-0.00091700	2.27076500
C	-6.22820300	-0.00096200	2.25359600
C	-6.90813800	-0.00049700	1.02733100
N	-6.30263800	0.00000800	-0.15727300
H	-4.28947500	-0.00127400	3.20791100
H	-6.79533300	-0.00135500	3.17891000
H	-7.99619700	-0.00053800	1.01472200
C	-4.08418300	0.00058400	-1.31500900
C	-2.68775400	0.00038500	-0.78462000
C	-2.71650300	-0.00023200	0.72448600
H	-2.21156600	-0.87908600	1.14321400
H	-2.21160200	0.87829900	1.14393600
O	-4.42013800	0.00108600	-2.48485700
C	-1.62736600	0.00070600	-1.59908600
H	-1.82953400	0.00113400	-2.67161900
C	-0.21759800	0.00050800	-1.14615900
C	0.45172300	1.20788300	-0.93442000
C	1.78567800	1.23065400	-0.51099200
C	2.46607200	0.00012000	-0.29492900
C	1.78560100	-1.23021600	-0.51186800
C	0.45165000	-1.20706000	-0.93528600
C	2.49398600	2.45790300	-0.28544100
C	3.82167800	-0.00007500	0.13570600
C	4.50012600	1.23238100	0.35160300
C	3.78887200	2.45770900	0.12582200
C	5.83488400	1.20798000	0.77551300
H	6.35524800	2.14766900	0.94079600
C	6.49152800	-0.00046000	0.98358200
C	5.83480500	-1.20871100	0.77466400
C	4.50004500	-1.23272800	0.35073700
C	3.78871200	-2.45785000	0.12409300
C	2.49382800	-2.45767000	-0.28717800
H	1.96943500	-3.39464200	-0.45509700

H	4.31228000	-3.39594700	0.28900200
H	1.96965400	3.39502700	-0.45270000
H	-0.06878100	2.14719100	-1.10207500
H	-0.06891200	-2.14621600	-1.10361200
H	4.31250400	3.39565500	0.29138300
H	7.52669300	-0.00060900	1.31198100
H	6.35510800	-2.14855000	0.93928700

- **1**-Pl$_c$

0 1

C	1.87626600	-0.46946100	-0.00006900
C	2.25171500	0.87413500	-0.00004200
C	3.60832100	1.17963500	0.00004400
C	4.49838900	0.11170500	0.00014700
C	4.00639400	-1.20042800	0.00018200
N	2.71172100	-1.51048000	0.00006300
H	3.96336300	2.20654700	0.00003000
H	5.57039400	0.28097900	0.00019300
H	4.70321000	-2.03646200	0.00035500
C	0.39376200	-0.58119800	-0.00017900
C	-0.11871700	0.78854600	-0.00017100
C	1.04492600	1.76155000	-0.00002400
H	1.03372200	2.41726600	-0.88018500
H	1.03359500	2.41700400	0.88034000
O	-0.22155800	-1.65449300	-0.00027000
C	-1.41761900	1.21644300	-0.00010800
H	-1.55998300	2.29714400	0.00003100
C	-2.64452900	0.50478600	-0.00009000
C	-3.94316100	1.03649900	0.00012600
C	-4.83899300	-0.03829200	0.00022000
C	-4.07341800	-1.20295200	0.00020100
N	-2.77077600	-0.86493900	-0.00013900
H	-1.93425000	-1.46507700	-0.00007000
H	-4.18246600	2.09161800	0.00007600
H	-5.91891300	0.00760800	0.00028100
H	-4.38285300	-2.23856400	0.00021700

- **1**-Pl$_t$

0 1

C	2.17872900	0.51386000	0.00000500
C	1.78753300	-0.82537000	0.00011700
C	2.77605700	-1.80248000	0.00014900
C	4.09714300	-1.36833300	0.00012900
C	4.37401100	0.00446200	0.00007300

N	3.43879500	0.95298900	0.00000600
H	2.53388000	-2.86185500	0.00020200
H	4.91652200	-2.07997700	0.00014300
H	5.40747400	0.34552300	0.00008500
C	0.96959400	1.38888700	-0.00015200
C	-0.18857400	0.47327300	-0.00011400
C	0.28808200	-0.95848000	0.00005800
H	-0.05180200	-1.51466300	0.88523000
H	-0.05172400	-1.51491400	-0.88497500
O	0.95015700	2.61110700	-0.00030000
C	-1.44938500	0.96798300	-0.00002200
H	-1.51813900	2.05550500	-0.00008600
C	-2.70389500	0.29186900	0.00002100
C	-3.98030400	0.85697900	0.00044500
C	-4.91979800	-0.18783500	0.00035200
C	-4.20556200	-1.37427100	-0.00036500
N	-2.88310800	-1.07620500	-0.00034400
H	-2.14450600	-1.75946000	-0.00086600
H	-4.18166300	1.91967300	0.00089800
H	-5.99684800	-0.09986400	0.00077600
H	-4.54605300	-2.39957000	-0.00082600

- **1**-Pl_{cc}^{ts}

0 1

C	-1.93760100	0.52496900	-0.01046100
C	-1.93365100	-0.84794200	-0.27456300
C	-3.09052500	-1.57415300	-0.05165000
C	-4.20145600	-0.87581400	0.42901100
C	-4.09459800	0.49445600	0.65936100
N	-2.98231300	1.21015600	0.44947600
H	-3.14094000	-2.64380900	-0.24150400
H	-5.14013600	-1.38520300	0.62314200
H	-4.95545500	1.04634900	1.03461600
C	-0.59402200	1.13118600	-0.33092900
C	0.19812100	0.02262700	-0.72950300
C	-0.58764300	-1.27248100	-0.77656000
H	-0.66847500	-1.69523900	-1.79107000
H	-0.16258700	-2.07217500	-0.14988300
O	-0.32667100	2.34142500	-0.21611700
C	1.54704500	0.10538000	-1.21938300
H	1.77404700	0.32146300	-2.27165800
C	2.64426300	-0.04846800	-0.41446500
C	4.04677900	-0.04093200	-0.68403200
C	4.70382500	-0.24725900	0.50719000

C	3.71235400	-0.36849800	1.51161500
N	2.51639100	-0.25866800	0.95580300
H	1.60755100	-0.26002100	1.40576500
H	4.48156400	0.09756400	-1.66438700
H	5.76933400	-0.30264300	0.67679200
H	3.83257100	-0.51652500	2.57639700

- **1**-Pl_{ct}^{ts}

0 1

C	-1.93789900	0.52491400	-0.01019100
C	-1.93380700	-0.84790800	-0.27476200
C	-3.09067900	-1.57426200	-0.05230500
C	-4.20170300	-0.87615900	0.42848600
C	-4.09493800	0.49402000	0.65942500
N	-2.98267800	1.20987700	0.44992000
H	-3.14102300	-2.64384500	-0.24259000
H	-5.14039300	-1.38566500	0.62226300
H	-4.95585400	1.04570200	1.03485400
C	-0.59438300	1.13137500	-0.33044600
C	0.19810700	0.02293300	-0.72861500
C	-0.58766000	-1.27215500	-0.77665200
H	-0.66828600	-1.69433700	-1.79142100
H	-0.16265100	-2.07215900	-0.15034600
O	-0.32736400	2.34168400	-0.21569500
C	1.54691500	0.10609200	-1.21876600
H	1.77366100	0.32263500	-2.27101000
C	2.64445300	-0.04812700	-0.41427300
C	4.04685000	-0.04046600	-0.68443400
C	4.70441000	-0.24744400	0.50639200
C	3.71337400	-0.36918700	1.51118000
N	2.51716800	-0.25916600	0.95592300
H	1.60852500	-0.26006300	1.40629200
H	4.48121700	0.09845300	-1.66491400
H	5.76998900	-0.30307700	0.67547800
H	3.83405500	-0.51783300	2.57582400

- **1**-Pl_{tt}^{ts}

0 1

C	2.18115100	0.46841800	-0.00432700
C	1.69636300	-0.84067600	0.01210200
C	2.62061900	-1.88150600	0.01378000
C	3.96599400	-1.53635200	-0.00146600
C	4.33678000	-0.18405600	-0.01824700
N	3.46842300	0.82361800	-0.01998200

H	2.30821100	-2.92194100	0.02689400
H	4.73586300	-2.30130100	-0.00051600
H	5.39096300	0.08524600	-0.03066300
C	1.03969800	1.42372000	-0.00028900
C	-0.19340100	0.57752000	0.02470600
C	0.19242800	-0.88113600	0.02432100
H	-0.19705200	-1.40289200	0.90635600
H	-0.20460000	-1.40996800	-0.85039600
O	1.08806500	2.63947100	-0.01399400
C	-1.41766400	1.11601700	0.06010100
H	-1.47303300	2.20635300	0.06904400
C	-2.66446200	0.33671900	0.09735900
C	-3.42001900	-0.11195300	1.16295300
C	-4.54078500	-0.80354000	0.63125800
C	-4.44001000	-0.75493000	-0.73985500
N	-3.30173800	-0.06477300	-1.04921900
H	-2.97905300	0.13673800	-1.98164800
H	-3.18418900	0.04724500	2.20614300
H	-5.33080700	-1.28373700	1.19205600
H	-5.07775300	-1.15289000	-1.51529200

Table S2. S_1 geometries of the cis- and trans- structures under study at the PBE0/def2-tzvp level of theory in chloroform solvent.

- **1**-An_c

0 1

C	-4.01489500	0.02905400	-0.40287700
C	-4.20826000	-1.13119600	0.36061900
C	-5.45800800	-1.70577300	0.38919300
C	-6.46701900	-1.08568000	-0.35059800
C	-6.16934200	0.06360900	-1.07353100
N	-4.96707500	0.63306400	-1.11749400
H	-5.65786400	-2.60504100	0.96261900
H	-7.47309200	-1.48643000	-0.36796500
H	-6.95093900	0.55134400	-1.65065100
C	-2.61404600	0.44934300	-0.31553600
C	-1.95536700	-0.47348200	0.59161700
C	-2.93105700	-1.53104600	1.03024900
H	-2.60883000	-2.53202000	0.71931700
H	-3.03895800	-1.56277400	2.11990500
O	-2.08130600	1.40108600	-0.92206900
C	-0.63503500	-0.43515200	0.94474900

H	-0.23131600	-1.30290000	1.45862600
C	0.25556200	0.66953500	0.69446800
C	1.64128900	0.46516600	0.38783200
C	-0.21232000	1.99603800	0.77841600
C	2.19660800	-0.80728000	0.28407400
C	2.48804400	1.60083800	0.14279400
C	0.61732900	3.08584000	0.51910000
H	-1.21985700	2.16924200	1.12455800
C	3.55011600	-1.01429900	-0.02370300
H	1.57138500	-1.67861600	0.43766400
C	3.83014300	1.39878200	-0.15896800
C	1.94251200	2.90362900	0.20276200
H	0.21112700	4.08782000	0.59278400
C	4.10151000	-2.30803500	-0.12384500
C	4.39124800	0.11668700	-0.24393000
H	4.46325200	2.26187800	-0.34012600
H	2.59033600	3.75282100	0.01583900
C	5.43132300	-2.47985900	-0.42329500
H	3.45983200	-3.16691800	0.03973500
C	5.75223500	-0.09199500	-0.54741400
C	6.26054800	-1.36535700	-0.63476100
H	5.84554000	-3.47821600	-0.49749700
H	6.39275200	0.76764200	-0.71094900
H	7.30789400	-1.51416200	-0.86934700

- **1**-An_t

0 1

C	-4.19836000	-0.94131700	0.02202600
C	-4.32648800	0.44743100	-0.04683700
C	-5.58783400	0.99889200	-0.12587800
C	-6.66821200	0.12048700	-0.13320000
C	-6.43140100	-1.25005000	-0.06141500
N	-5.22323200	-1.79755700	0.01598000
H	-5.73927300	2.07205200	-0.18067400
H	-7.68574600	0.48709700	-0.19390700
H	-7.27189800	-1.93958600	-0.06730000
C	-2.77281300	-1.30679500	0.09850500
C	-2.02387800	-0.04861500	0.06402900
C	-2.98148400	1.10686600	-0.01775300
H	-2.89947100	1.78101200	0.84358600
H	-2.82417900	1.72092600	-0.91256200
O	-2.29946700	-2.44387600	0.17247700
C	-0.65311600	-0.09144400	0.09001600
H	-0.29111500	-1.11200400	0.14157500

C	0.31345800	0.95171200	0.05904000
C	1.72732900	0.64305800	0.03046000
C	-0.03736900	2.32330000	0.05753500
C	2.21193400	-0.66363400	0.00904800
C	2.69038100	1.70870000	0.01462900
C	0.90879100	3.33090200	0.03935000
H	-1.07975600	2.60029300	0.08061600
C	3.58563600	-0.97472700	-0.01864900
H	1.52453200	-1.49900700	0.00767900
C	4.06031000	1.39935200	-0.00991300
C	2.26721800	3.04663600	0.02034500
H	0.57891000	4.36373600	0.04309800
C	4.04111500	-2.29988600	-0.03954400
C	4.53565000	0.08626300	-0.02593600
H	4.77414400	2.21734600	-0.01826700
H	3.00414800	3.84042200	0.00839800
C	5.39762200	-2.57751700	-0.06550500
H	3.31819700	-3.10788100	-0.03470200
C	5.90943000	-0.22282600	-0.05210100
C	6.33057600	-1.53760700	-0.07146500
H	5.73659000	-3.60610000	-0.08109200
H	6.63275500	0.58506000	-0.05723500
H	7.38955200	-1.76455200	-0.09162000

- **1**-Na_c

0 1

C	-3.49955100	0.47895000	-0.00012000
C	-3.80061300	-0.88686400	0.00009100
C	-5.11681700	-1.28634300	0.00034600
C	-6.08809400	-0.28171200	0.00044200
C	-5.68513700	1.04927800	0.00030700
N	-4.41751800	1.45343300	-0.00000700
H	-5.39563400	-2.33501100	0.00045600
H	-7.14377300	-0.52414300	0.00062800
H	-6.43753600	1.83447600	0.00035800
C	-2.04874400	0.69031500	-0.00039500
C	-1.43880800	-0.64364500	-0.00016900
C	-2.53606100	-1.68045700	-0.00006500
H	-2.46639400	-2.33454100	-0.87753300
H	-2.46620200	-2.33455600	0.87737900
O	-1.48872000	1.80147500	-0.00034400
C	-0.13109100	-1.08155100	-0.00005600
H	-0.02745500	-2.16483100	0.00011100
C	1.13438600	-0.40116800	-0.00008100

C	2.31304300	-1.19323500	0.00002100
C	3.59411300	-0.64137800	0.00004900
C	3.74346900	0.77326500	-0.00003900
C	2.56758100	1.56111200	-0.00012200
C	1.30847900	0.99642600	-0.00010600
C	4.75735700	-1.44288000	0.00013300
C	5.02341400	1.32957300	-0.00008200
C	6.15806100	0.51616100	-0.00000600
C	6.01922600	-0.86650900	0.00010300
H	4.65286200	-2.52237400	0.00020400
H	2.21455100	-2.27470000	0.00010900
H	0.41760700	1.61760500	-0.00018900
H	6.89825200	-1.49933600	0.00016400
H	2.66604300	2.64164900	-0.00022600
H	5.13139300	2.40868500	-0.00015500
H	7.14339100	0.96520500	-0.00001700

- **1**-Na_t

0 1

C	3.73804000	0.51774600	-0.00000400
C	3.36933700	-0.83387400	0.00004500
C	4.34821200	-1.79859700	0.00019300
C	5.67640800	-1.36041400	0.00027400
C	5.93871800	0.00573900	0.00019000
N	5.00559000	0.95318400	0.00003900
H	4.10803700	-2.85678200	0.00024100
H	6.49792600	-2.06622100	0.00038000
H	6.96998300	0.35089500	0.00023200
C	2.54561300	1.36140500	-0.00011000
C	1.39219300	0.44470000	-0.00012300
C	1.87497700	-0.97135900	-0.00009600
H	1.52508500	-1.52760300	0.87890000
H	1.52525200	-1.52763500	-0.87913400
O	2.47833500	2.59669700	-0.00030900
C	0.11357600	0.95472500	-0.00019800
H	0.07813900	2.04159500	-0.00022800
C	-1.14452500	0.29100400	-0.00011400
C	-2.32514900	1.09099700	0.00003700
C	-3.60078200	0.54446100	0.00004800
C	-3.75424000	-0.87351100	-0.00003800
C	-2.57915600	-1.67105800	-0.00015500
C	-1.32830400	-1.11417700	-0.00020000
C	-4.76752800	1.35431700	0.00017200
C	-5.03442400	-1.42272300	0.00000300

C	-6.16311900	-0.60422800	0.00012900
C	-6.02331800	0.78355100	0.00021500
H	-4.65609900	2.43303900	0.00023600
H	-2.21376900	2.17061900	0.00011500
H	-0.47057700	-1.76954400	-0.00032200
H	-6.90295600	1.41559300	0.00030900
H	-2.68645000	-2.75053600	-0.00022300
H	-5.14946300	-2.50114300	-0.00006300
H	-7.15009000	-1.05036300	0.00015600

- **1**-Ph_c

0 1

C	-3.12348100	0.54193500	-0.34193100
C	-4.16671600	-0.19507300	0.24696800
C	-5.42574600	0.35589800	0.27597100
C	-5.58680300	1.62459200	-0.28571700
C	-4.48671600	2.26688700	-0.84130700
N	-3.25911600	1.75362800	-0.88381400
H	-6.26558200	-0.16923200	0.71862700
H	-6.55386500	2.11188400	-0.29323900
H	-4.60637000	3.25494700	-1.27793900
C	-1.90416500	-0.24619700	-0.29200500
C	-2.19412900	-1.46851800	0.40233200
C	-3.65324900	-1.50595900	0.75680500
H	-4.15828500	-2.35369800	0.27967800
H	-3.81261000	-1.61307200	1.83471200
O	-0.77724100	0.07401200	-0.78256600
C	-1.25098400	-2.43490400	0.62865700
C	3.73726400	3.27059800	0.48283200
C	4.48997300	2.21709600	0.03404500
C	3.92861500	0.93138900	-0.09586900
C	2.56645700	0.72382800	0.25454700
C	1.81348900	1.83221200	0.68771400
C	2.38501500	3.07454100	0.80428300
C	4.70310900	-0.15349700	-0.58611500
C	2.00807900	-0.59593400	0.13182300
C	2.81529100	-1.64442600	-0.36181400
C	4.16317100	-1.39428900	-0.72173600
C	2.28375600	-2.95558000	-0.42943700
H	2.92355600	-3.75242900	-0.79342500
C	0.99010300	-3.23065400	-0.06195600
C	0.14273900	-2.20378100	0.37569900
C	0.68649200	-0.89558500	0.52805300
H	5.73828300	0.02839400	-0.85413500

H	4.17829800	4.25610100	0.57775700
H	5.53161500	2.35857500	-0.23391900
H	0.75961300	1.71427700	0.90326500
H	1.78399400	3.91293500	1.13687800
H	4.76316000	-2.21792700	-1.09341700
H	0.60080800	-4.23903000	-0.14781000
H	0.17999800	-0.21798100	1.19920500
H	-1.56025500	-3.42872400	0.93992700

- **1**-Ph_t

0 1

C	4.34621600	-0.06749700	-0.00000200
C	3.47836200	1.03042900	0.00003900
C	4.00412800	2.30229500	0.00008400
C	5.39509800	2.41719900	0.00008600
C	6.17172800	1.26118900	0.00004300
N	5.68213400	0.02606900	-0.00000200
H	3.36984100	3.18274900	0.00011500
H	5.87604500	3.38770400	0.00012200
H	7.25573000	1.34681400	0.00004400
C	3.57644300	-1.31338300	-0.00004000
C	2.15958300	-0.92100600	-0.00002200
C	2.04977800	0.57445800	0.00002300
H	1.51462200	0.95213900	-0.88011200
H	1.51461300	0.95208900	0.88017200
O	4.00471100	-2.47212600	-0.00009300
C	1.18585800	-1.89556600	-0.00003800
C	-4.62777500	3.09661300	-0.00004800
C	-5.25796100	1.87750600	0.00005200
C	-4.51412900	0.67373700	0.00005600
C	-3.09035400	0.72207000	-0.00003100
C	-2.48290000	1.97531100	-0.00014600
C	-3.23238800	3.14310300	-0.00015300
C	-5.17403600	-0.56680900	0.00012500
C	-2.33596800	-0.52809300	-0.00001200
C	-3.06345800	-1.75910100	0.00002500
C	-4.45886800	-1.75233900	0.00009500
C	-2.34016900	-2.98685500	0.00001300
H	-2.90123700	-3.91496400	0.00002800
C	-0.98281900	-3.00320800	-0.00001500
C	-0.22879500	-1.79402900	-0.00002200
C	-0.95495100	-0.57180600	-0.00001600
H	-6.25786800	-0.58689700	0.00019000
H	-5.20415100	4.01356600	-0.00005100

H	-6.34080300	1.81612300	0.00012500
H	-1.40402900	2.06021300	-0.00024500
H	-2.72373800	4.09994500	-0.00024400
H	-4.98794200	-2.69894700	0.00013500
H	-0.44707500	-3.94547500	-0.00002600
H	-0.39756500	0.35109400	-0.00000600
H	1.58519000	-2.90693000	-0.00006100

- **1**-Pn_c

C	4.64805100	-0.58414900	-0.00013800
C	5.16542300	0.71474500	-0.00011200
C	6.52926600	0.89584200	0.00001300
C	7.32683600	-0.25115400	0.00013000
C	6.71461000	-1.49800400	0.00011400
N	5.39669500	-1.69171100	-0.00001900
H	6.97365900	1.88606700	0.00003800
H	8.40780400	-0.18165900	0.00024100
H	7.32955600	-2.39507500	0.00020700
C	3.17666000	-0.55644900	-0.00038200
C	2.79099700	0.84443000	-0.00023500
C	4.04305600	1.69741100	-0.00020500
H	4.08436300	2.35240700	-0.87829300
H	4.08428200	2.35244700	0.87785700
O	2.45872800	-1.58091200	-0.00013000
C	1.57284300	1.48545200	-0.00011200
H	1.63506900	2.57167700	-0.00002700
C	0.21017300	1.00682800	-0.00006400
C	-0.82957900	1.94496300	0.00006900
C	-2.19230200	1.58752200	0.00012500
C	-2.54852600	0.21935600	0.00004400
C	-1.51753000	-0.74956400	-0.00008600
C	-0.16977200	-0.34799400	-0.00013300
C	-3.22395000	2.55219300	0.00025800
C	-3.89649000	-0.16876200	0.00008900
C	-4.91660800	0.81228100	0.00021900
C	-4.54669600	2.17615500	0.00030500
C	-6.26399200	0.39603600	0.00025800
H	-7.04447200	1.14881100	0.00035500
C	-6.59741000	-0.94878400	0.00017200
C	-5.59911900	-1.90974600	0.00004600
C	-4.23725900	-1.54397400	0.00000100
C	-3.19893900	-2.50183800	-0.00012900
C	-1.87770900	-2.11596200	-0.00017200
H	-1.09303700	-2.86361600	-0.00027400

H	-3.45515200	-3.55523000	-0.00019700
H	-2.96171900	3.60424300	0.00032400
H	-0.58297900	3.00277500	0.00013300
H	0.62370700	-1.09480100	-0.00023900
H	-5.32438000	2.93162500	0.00040700
H	-7.63747300	-1.24866200	0.00020400
H	-5.85812300	-2.96274500	-0.00002100

- **1**-Pn_t

0 1

C	-5.06187500	0.33402000	0.00000400
C	-4.47879000	-0.93798200	-0.00004900
C	-5.29002400	-2.04893700	-0.00002600
C	-6.67042400	-1.83414600	0.00006000
C	-7.14991000	-0.52991900	0.00011900
N	-6.38037100	0.55659000	0.00009200
H	-4.88132700	-3.05451700	-0.00006500
H	-7.36626300	-2.66423200	0.00008700
H	-8.22340300	-0.35525700	0.00018700
C	-4.01490300	1.36647400	-0.00008400
C	-2.74122000	0.66051500	-0.00006700
C	-2.98459100	-0.82429200	-0.00010900
H	-2.55679500	-1.32045000	-0.88036600
H	-2.55672800	-1.32050500	0.88008500
O	-4.18521900	2.59633000	0.00010700
C	-1.56243100	1.35597000	-0.00003800
H	-1.68563800	2.43578000	-0.00002700
C	-0.20210800	0.89875900	-0.00002600
C	0.83132900	1.85470300	-0.00001800
C	2.19333700	1.52018600	-0.00000900
C	2.57527500	0.15587200	-0.00000600
C	1.56195300	-0.83144200	-0.00001100
C	0.20868200	-0.44764300	-0.00001900
C	3.20928200	2.50397400	-0.00000500
C	3.92927900	-0.20677900	0.00000100
C	4.93209300	0.79359000	0.00000500
C	4.53792800	2.15126000	0.00000100
C	6.28667400	0.40362000	0.00001200
H	7.05193700	1.17191000	0.00001500
C	6.64774500	-0.93545200	0.00001700
C	5.66839700	-1.91418100	0.00001400
C	4.29823400	-1.57433800	0.00000600
C	3.27885600	-2.55103300	0.00000300
C	1.95086100	-2.19057800	-0.00000600

H	1.18496000	-2.95793700	-0.00000900
H	3.55426300	-3.59961200	0.00000700
H	2.92778600	3.55097000	-0.00000800
H	0.56241200	2.90647500	-0.00002000
H	-0.53084300	-1.23673200	-0.00002000
H	5.30171700	2.92062800	0.00000400
H	7.69350300	-1.21443200	0.00002300
H	5.94689200	-2.96218300	0.00001700

- **1**-Pl_c

0 1

C	1.84327200	-0.47643000	0.00002800
C	2.23541700	0.87142800	-0.00001000
C	3.57331300	1.18249300	-0.00001100
C	4.47665600	0.11110000	0.00002600
C	3.98610900	-1.19256200	0.00006100
N	2.69849200	-1.51626300	0.00006300
H	3.92159000	2.20971100	-0.00003800
H	5.54607000	0.28231000	0.00002600
H	4.68735400	-2.02394500	0.00008700
C	0.40477400	-0.58999600	0.00002600
C	-0.13177200	0.80382100	-0.00001800
C	1.02882700	1.75822800	-0.00003700
H	0.99885700	2.41517300	-0.87703300
H	0.99885000	2.41521300	0.87692900
O	-0.24906400	-1.65539000	0.00009000
C	-1.42252300	1.27128200	-0.00003700
H	-1.55402900	2.34877600	-0.00004600
C	-2.63575300	0.52843100	-0.00004000
C	-3.97206200	1.01257600	-0.00003000
C	-4.80722900	-0.08903900	0.00008200
C	-3.98093300	-1.22897100	-0.00010500
N	-2.69875600	-0.83571200	-0.00008800
H	-1.80587000	-1.39498200	-0.00008100
H	-4.25190300	2.05502000	-0.00001300
H	-5.88594200	-0.09971400	0.00017300
H	-4.24919200	-2.27478100	-0.00015000

- **1**-Pl_t

0 1

C	2.16187900	0.53410100	-0.00003300
C	1.77632300	-0.81875600	-0.00007900
C	2.73619200	-1.79809600	0.00004900
C	4.07658900	-1.37825100	0.00022200

C	4.35764800	-0.01449900	0.00023800
N	3.44634800	0.95096200	0.00010300
H	2.47867000	-2.85199600	0.00001700
H	4.88785200	-2.09543600	0.00032800
H	5.39569600	0.31141700	0.00035400
C	0.99760700	1.38766400	-0.00017700
C	-0.18752500	0.47700700	-0.00017800
C	0.27692300	-0.94429000	-0.00028700
H	-0.07577900	-1.49183300	0.88393400
H	-0.07552600	-1.49166600	-0.88471200
O	0.90695700	2.61871100	-0.00019600
C	-1.45720200	0.99550800	-0.00015100
H	-1.52086100	2.07950700	-0.00009000
C	-2.68393200	0.29903500	-0.00001300
C	-3.99639500	0.84074800	0.00047700
C	-4.89358900	-0.21115100	0.00031000
C	-4.14404800	-1.39358400	0.00002200
N	-2.83633600	-1.07091000	-0.00026900
H	-2.08380100	-1.73768400	-0.00067500
H	-4.21564500	1.89716800	0.00079900
H	-5.97067800	-0.15937300	0.00049400
H	-4.46849300	-2.42277300	-0.00011400
